# STAG2 expression imparts distinct therapeutic vulnerabilities in muscle-invasive bladder cancer cells

**DOI:** 10.1038/s41389-025-00548-3

**Published:** 2025-03-01

**Authors:** Sarah R. Athans, Henry Withers, Aimee Stablewski, Katerina Gurova, Joyce Ohm, Anna Woloszynska

**Affiliations:** 1 Department of Pharmacology and Therapeutics, Roswell Park Comprehensive Cancer Center, Buffalo, NY USA; 2 Department of Biostatistics and Bioinformatics, Roswell Park Comprehensive Cancer Center, Buffalo, NY USA; 3 Department of Molecular and Cellular Biology, Roswell Park Comprehensive Cancer Center, Buffalo, NY USA; 4 Department of Cell Stress Biology, Roswell Park Comprehensive Cancer Center, Buffalo, NY USA; 5 Department of Cancer Genetics and Genomics, Roswell Park Comprehensive Cancer Center, Buffalo, NY USA

**Keywords:** Targeted therapies, High-throughput screening, Bladder cancer

## Abstract

Expression of stromal antigen 2 (STAG2), a member of the cohesin complex, is associated with aggressive tumor characteristics and worse clinical outcomes in muscle invasive bladder cancer (MIBC) patients. The mechanism by which STAG2 acts in a pro-oncogenic manner in bladder cancer remains unknown. Due to this elusive role of STAG2, targetable vulnerabilities based on STAG2 expression have not yet been identified. In the current study, we sought to uncover therapeutic vulnerabilities of muscle invasive bladder cancer cells based on the expression of STAG2. Using CRISPR-Cas9, we generated isogenic STAG2 wild-type (WT) and knock out (KO) cell lines and treated each cell line with a panel of 312 anti-cancer compounds. We identified 100 total drug hits and found that STAG2 KO sensitized cells to treatment with PLK1 inhibitor rigosertib, whereas STAG2 KO protected cells from treatment with MEK inhibitor TAK-733 and PI3K inhibitor PI-103. After querying drug sensitivity data of over 4500 drugs in 24 bladder cancer cell lines from the DepMap database, we found that cells with less STAG2 mRNA expression are more sensitive to ATR and CHK inhibition. In dose-response studies, STAG2 KO cells are more sensitive to the ATR inhibitor berzosertib, whereas STAG2 WT cells are more sensitive to PI3K inhibitor PI-103. These results, in combination with RNA-seq analysis of STAG2-regulated genes, suggest a novel role of STAG2 in regulating PI3K signaling in bladder cancer cells. Finally, synergy experiments revealed that berzosertib exhibits significant synergistic cytotoxicity in combination with cisplatin against MIBC cells. Altogether, our study presents evidence that berzosertib, PI-103, and the combination of berzosertib with cisplatin may be novel opportunities to investigate as precision medicine approaches for MIBC patients based on STAG2 tumor expression.

## Introduction

As a member of the cohesin complex, stromal antigen 2 (STAG2) functions in sister chromatid cohesion [1], DNA damage response via homologous recombination [2–4], and genome organization [5–8]. STAG2 is often mutated in bladder cancer, with a higher frequency of mutations in non-muscle invasive bladder cancer (NMIBC) than in muscle invasive bladder cancer (MIBC) [9]. Non-mutant *STAG2* is an independent predictor of progression from NMIBC to MIBC [10], and is associated with higher stage and higher-grade disease [11]. Conversely, loss-of-function mutations in *STAG2* are associated with better outcomes [11]. We have also shown that high tumor STAG2 protein expression, specifically in MIBC patients, is associated with significantly worse survival outcomes and an invasive phenotype [12]. Together, these data imply that STAG2 acts in an oncogenic manner in bladder cancer. This is contrary to the tumor suppressive role of STAG2 described in other cancer types such as Ewing sarcoma, acute myeloid leukemia, and pancreatic cancer [2, 13–15]. The pro-tumorigenic contributions of STAG2 in bladder cancer underlie the importance of identifying therapeutic vulnerabilities of STAG2 expressing cells to develop novel precision therapies for bladder cancer patients.

To date, several studies have identified vulnerabilities of STAG2 deficient or mutant cancer cells. STAG2 deficient glioblastoma and Ewing sarcoma cells are more sensitive to DNA alkylating agents, DNA crosslinking agents, topoisomerase inhibitors, Poly (ADP-ribose) polymerase (PARP) inhibitors, and ataxia telangiectasia and Rad3-related protein (ATR) inhibitors [16, 17]. In acute myeloid leukemia, STAG2 knock out (KO) or loss-of-function mutation sensitizes cancer cells to treatment with the PARP inhibitor talazoparib [2, 18]. We have previously shown that STAG2 loss increases the effects of cisplatin treatment in MIBC cells [12]. Conversely, no studies have identified a specific targetable vulnerability in cases of high STAG2 expression, which is particularly urgent in bladder cancer where STAG2 acts in an oncogenic manner [12].

In the current study, we sought to identify therapeutic vulnerabilities of MIBC cells based on STAG2 expression. Based on results of high throughput compound screening with a well annotated library of drugs, we characterize STAG2-dependent signaling pathways that confer sensitivity and may promote bladder cancer.

We determined differential STAG2-dependent sensitivities to a library of drugs via in vitro screening of isogenic MIBC cell lines with and without STAG2 knockout (KO). Our customized drug screening panel consisted of experimental and FDA-approved compounds with well characterized targets in cancer-related pathways such as PI3K, MEK, mTOR, and DNA damage response. We expanded our drug candidate search by performing additional in silico analysis of drug sensitivity, genomic, and transcriptomic data available for several bladder cancer cell lines in the Dependency Map (DepMap) portal, and candidate compounds were validated by dose-response testing using seven candidate drugs which emerged from our screening approach. We demonstrated that MIBC cells with STAG2 KO are more sensitive to the ATR inhibitor berzosertib, in line with the known function of STAG2 in DNA damage response [2–4]. We identified a novel synergistic relationship with berzosertib, olaparib, and talazoparib in combination with first line MIBC therapy cisplatin. Finally, we identified a therapeutic vulnerability of STAG2 wild-type cells to PI3K inhibitor PI-103. Taken together, these findings provide the groundwork for further investigation of berzosertib, olaparib, and PI-103 as precision therapy approaches in MIBC.

## Methods

### Cell culture

T24 cells (ATCC, catalog no. HTB-4) and cultured in McCoy’s 5 A media (Corning) with 10% FBS (Corning) and 1x Penicillin/Streptomycin (Corning). TCCSUP cells (ATCC, catalog no. HTB-5) were cultured in MEM media (Corning) with 10% FBS (Corning) and 1x Penicillin/Streptomycin (Corning). HB-CLS-1 (CLS, catalog no. 300190/p466_HB-CLS-1) were cultured in RPMI1640 (Corning) with 10% FBS and 1x penicillin/streptomycin (Corning). BO1 cells, generated previously [19], were cultured in a 3:1 (v:v) ratio of F12K (ATCC):DMEM (Corning) with 5% FBS and 1x penicillin/streptomycin. All cells were maintained in 5% carbon dioxide at 37 degrees Celsius. Cells were tested for *Mycoplasma* via MycoAlert *Mycoplasma* Detection Kit (Lonza, catalog no. LT07-218) after thawing and minimally once every 3 months thereafter. Cell lines were tested at least yearly by STR profiling. All experiments were performed with cells passaged less than 20 times after thaw.

### CRISPR-Cas9 Knockout (KO) of STAG2

Two single-guide RNA (sgRNA) sequences were designed using the CRISPOR (tetof.net) prediction website. These guides were used to target human STAG2 exons 5 and 8 in T24 and TCCSUP cells: TCTGGTCCAAACCGAATGAA TGG (e5), GATTATCCACTTACCATGGC TGG (e8). CRISPR RNA (crRNA) and tracer RNA (trRNA) were purchased from IDT DNA Technologies (Coralville, IA) and resuspended to 160 μM each. crRNA and trRNA (1:1) were complexed using touchdown polymerase chain reaction (PCR) (IDT DNA Technologies), and Cas9 3NLS protein (61uM) (IDT DNA Technologies) was added to make functional ribonucleoprotein (RNP). RNPs for both exons 5 and 8 were added to 3 × 10^6^ cells suspended in Opti-MEM medium (ThermoFisher Scientific). The cell mixture was electroporated using a NEPA21 electroporator (Bulldog Bio, Portsmouth, NH) and then transferred into 6-well plates containing complete medium without antibiotics (2 ml). Cells were single cell sorted into 96 well plates then expanded for knockout confirmation. T24 cells were analyzed for knockout by targeted next generation sequencing and deletion analysis by Washington University at St. Louis’ Genome Editing Core facility, and a large deletion was shown between exons 5 and 8. TCCSUP cells were screened for successful knockout of STAG2 protein by western blot.

### shRNA-Mediated Knock Down (KD) of STAG2

STAG2 was knocked down in HB-CLS-1 and BO1 cells as previously described [12]. Briefly, short hairpin RNAs (shRNA) targeting human STAG2, and a nontargeting control shRNA, were cloned into pGreenPuro shRNA Expression Lentivector (System Biosciences). The correct pGreenPuro shRNA constructs were verified by sequencing using H1 primer. 293TN cells (System Biosciences, catalog no. LV900A-1) were cultured to high confluence and co-transfected with 2 μg of the shSTAG2/nontargeting control lentiviral constructs and 10 μg of the pPACKH1-plasmid mix (System Biosciences, catalog no. LV500A-1) using Lipofectamine 2000 (Invitrogen, catalog no. 11668-027). The viral supernatant was collected at 48 and 72 h after transfection and 0.45 μm filtered. BO1 and HB-CLS-1 cells were infected with lentivirus in the presence of 8 μg/mL of polybrene (Santa Cruz Biotechnology, catalog no. SC-134220). Transduced cells were enriched by puromycin selection for 1 week, and knockdown of STAG2 expression verified by western blot.

### Western blotting

Cells were cultured to 75–80% confluence and lysed in Triton X-100/SDS lysis buffer (1% Triton X-100, 0.1% SDS, 50 mM Tris, 150 mM NaCl) containing protease and phosphatase inhibitors (Roche). Protein concentration was determined using Bio-Rad protein assay (Biorad, Cat no. 500-0116). Equal amounts of protein lysates (50 µg) were resolved on a 4–20% gradient SDS-PAGE and electro transferred onto PVDF membranes. Blots were incubated with STAG2 antibody (1:500, Cell Signaling Technology Cat no. 5882) and GAPDH antibody (1:10,000, Abcam Cat no. ab9485) overnight at 4 °C, washed three times with TBST, incubated for 1 h at room temperature with LICOR IRDye anti-Rabbit (1:4000) and LiCOR IRDye anti-mouse (1:5000) antibodies, washed three times with TBST, then detected and quantified on LICOR Odyssey Clx and ImageStudio software. GAPDH was used for assessment of protein loading and to report relative expression of STAG2.

### Proliferation assays

Cells were seeded in 96 well plates at a density of 500 (T24) and 1000 (TCCSUP) cells per well. Cell density was analyzed 24-, 48-, 72-, and 96-h time points using the sulforhodamine B colorimetric assay [20]. Briefly, cells were fixed for 1 h with 10% (v/v) trichloroacetic acid, stained for 30 min with 0.057% (w/v) sulforhodamine B, then washed four times with 1% (v/v) acetic acid. Dye was solubilized in 100 µL 10 mM Tris-base solution then read with a microplate reader at 510 nM. Percent cells in each well at each time point were normalized to the average reading for each cell line at the 24-h time point. Statistical analysis was performed at each time point comparing % cells to Control A6 (T24) or CC1 (TCCSUP) by one-way ANOVA with multiple comparisons.

### Drug screening

On day 1, cells were seeded at a density of 1500 cells/well in 40 µL of media in 384 well plates using a BioTek MicroFlo Select dispenser. On day 2, the compound library was added using a 384 well pin tool array (V&P Scientific) on a JANUS Robotic Liquid Handler (Perkin Elmer) A complete list of compounds can be found in Table S1. Plates were incubated for 72 h at 37 °C and 5% CO2. After incubation, 8 µL of 6x resazurin solution, diluted in the appropriate media from 36x stock, was added to each well. Plates were incubated for 4–6 h at 37 °C and 5% CO2 to allow cells to metabolize resazurin. Upon cellular conversion of resazurin to resorufin, fluorescence intensity was measured using Envision Multilabel plate reader (Perkin Elmer) (Excitation 570 nM, Emission 615 nM). Within-plate viability is calculated with the following equation:

$${Cell\; viability}\left( \% \right)=\,\displaystyle\frac{\left({Reading\; of\; experimental\; well}\right)-({Average\; reading\; of\; background\; wells}\left({media\; only}\right))}{\left({Average\; reading\; of\; untreated\; wells}\right)-\left({Average\; reading\; of\; backround\; well}({media\; only})\right)}* 100 \%$$

### DepMap analyses

The following datasets were downloaded from the DepMap database: drug sensitivity data (version 19Q4); gene expression and gene dependency data, (version 22Q1); CCLE damaging mutations. Bladder carcinoma cell lines (*n* = 36) were isolated for analysis. Cell lines were categorized into ‘STAG2-high’ or ‘STAG2-low’ groups based on the median mRNA expression of STAG2 (5.414 transcripts per million). Statistical comparisons, where appropriate, were made between groups using custom R scripts and the wilcox_test function within the rstatix package [21].

### Dose response testing and EC_50_ Calculations

Berzosertib, PI-103, olaparib, talazoparib, rigosertib, TAK-733, and prexasertib were purchased from MedChemExpress and stock solutions were prepared to a concentration of 10 mM in DMSO. Cells were seeded at a density of 500 cells/50 µL per well in clear 384 well plates. Cells were allowed to attach to the plate overnight. The next day, cells were treated with drugs using a D300e Digital Dispenser (Tecan). Plates were randomized and normalized to the highest-class volume of DMSO. Plates were incubated for 72 h in a 5% carbon dioxide, 37-degree Celsius incubator. After incubation, 10 µL of 6x resazurin solution, diluted in the appropriate media from 36x stock, was added to each well. Plates were incubated with resazurin for 6 h (T24 and TCCSUP cells) or 24 h (BO1 and HB-CLS-1 cells) then were read from the bottom of the plate using a microplate reader (BioTek Synergy H1, Agilent) with the following specifications: Excitation, 560 nm; Emission, 590 nm. Cell viability in each experimental well was calculated with the following equation for each individual plate:

$${Cell\; viability}\left( \% \right)=\,\displaystyle\frac{\left({Reading\; of\; experimental\; well}\right)-({Average\; reading\; of\; background\; wells}\left({media\; only}\right))}{\left({Average\; reading\; of\; untreated\; wells}\right)-\left({Average\; reading\; of\; backround\; well}({media\; only})\right)}* 100 \%$$

Cell viability values were utilized to create a 4-paramater log-logistic model of dose-response for each cell line-drug combination using the package drda [22] in R. Half maximal effective concentrations (EC_50_) values were reported from the model’s calculated effective_dose values. EC_50_ values for each cell line were divided by the EC_50_ average of control cell lines (T24 Control A6, TCCSUP CC1, BO1-Control, HB-CLS-1 Control) to calculate EC_50_ ratios, allowing for comparisons across experiments. Statistical analysis of EC_50_ ratios was performed by two-tailed t-tests or one-way ANOVA with multiple comparisons, as appropriate.

### Synergy analysis

Cisplatin (MedChemExpress) was prepared in water with 0.3% Tween-20 to a concentration of 3 mM. The addition of Tween-20 is required as a surfactant for compatibility with the D300e Digital Dispenser. Cells were seeded at 500 cells/50 µL per well in clear 384 well plates. Cells were allowed to attach to the plate overnight. The next day, cells were treated with drug combinations in a synergy design matrix using a D300e Digital Dispenser (Tecan). Plates were randomized and normalized to the highest-class volume of water + 0.3% Tween 20. Plates were incubated for 72 h in a 5% carbon dioxide, 37-degree Celsius incubator. Cell viability was calculated as described above for dose-response testing. Degree of synergy was evaluated and visualized using Bioconductor package synergyfinder in R [23].

## Results

### Differential expression of STAG2 drives distinct therapeutic vulnerabilities in MIBC cells

**Fig. 1:**
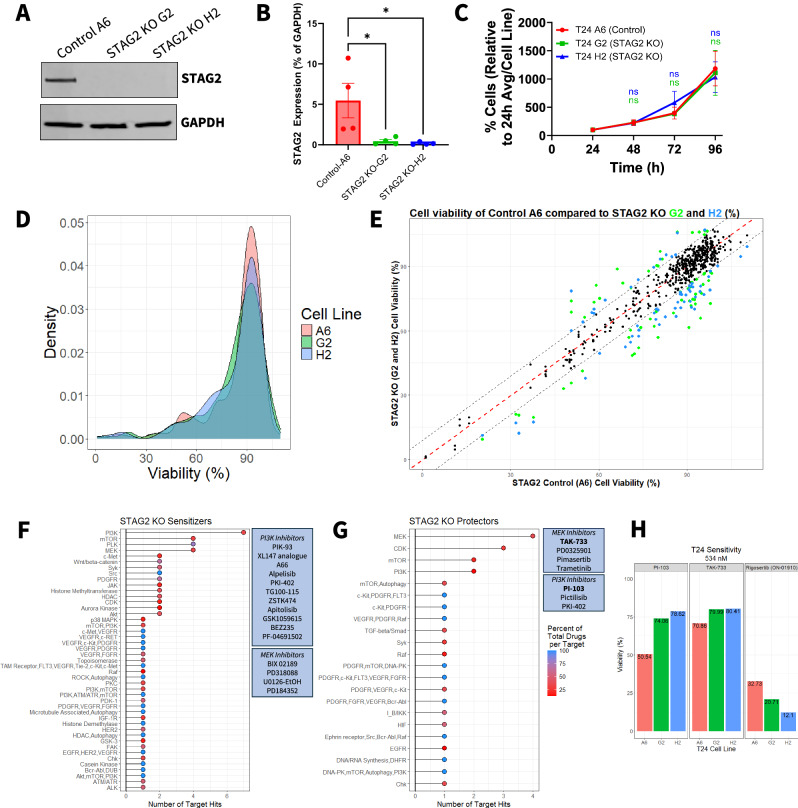
Differential expression of STAG2 induces distinct therapeutic vulnerabilities in MIBC cells. **A** Western blot of isogenic clonal T24 cell lines indicating complete knock out of STAG2 protein expression by CRISPR-Cas9 in STAG2 KO G2 and STAG2 KO H2 cell lines. Control A6 cell line maintains STAG2 expression. GAPDH used as a loading control. Images representative of four independent experiments. **B** Western blot quantification of four independent experiments showing the expression of STAG2 in control A6, STAG2 KO G2 and STAG2 KO H2 cell lines as a percentage of GAPDH expression. Statistics assessed via one-way ANOVA with multiple comparisons using Control A6 as a reference. *p < 0.05. **C** Cell number of T24 control A6, STAG2 KO G2, and STAG2 KO H2 cell lines over a period of 96 h measured via SRB assay every 24 h. Data is representative of three independent experiments and a minimum of five technical replicates. Presented as % cells relative to the average at 24 h for each cell line. Error bars represent SEM. Statistics assessed via one-way ANOVA with multiple comparisons using Control A6 as a reference. **D** Density plot showing distribution of cell viability after 72-h treatment across all drugs (*n* = 312) from the drug screen for each cell line. **E** Cell viability scatter plot of control A6 versus STAG2 KO G2 and STAG2 KO H2 for each drug in the drug screen. Each point represents viability after treatment with a single drug per comparison (A6 vs G2 and A6 vs H2). Red dotted line indicates the point at which a drug is equally cytotoxic to both the control and the STAG2 KO cell lines. Colored dots indicate drugs considered hits; green indicates hits when comparing viabilities of A6/G2, blue indicates hits when comparing viabilities of A6/H2. Hits determined as a viability difference between control and KO of greater than ±9%. **F** Cumulative number of times drugs targeting a specific molecule were classified as STAG2 KO sensitizer and **G** STAG2 KO protector drug hits in A6/G2 and A6/H2 comparisons. Color represents the number of drugs classified as hits for a target as a percentage of total drugs per target molecule included in the entire drug screen. Boxed callouts show dugs targeting PI3K or MEK in associated setting. **H** Percent viability of T24 A6 control, STAG2 KO G2, and STAG2 KO H2 after 72-h treatment with the indicated drugs at 534 nM derived from the drug screen. n.s.: not significant.

To model the effects of STAG2 loss in MIBC, we utilized CRISPR-Cas9 with gRNAs targeting exons 5 and 8 to knock out (KO) STAG2 expression in vitro in T24 MIBC cells, a cell line with relatively high endogenous STAG2 expression that has well characterized mutation and expression data publicly available [24]. We confirmed STAG2 KO in two clonal cell lines (STAG2 KO G2 and STAG2 KO H2) by western blot (Fig. 1A). The control cell line (Control A6) was generated by clonal selection of cells electroporated with gRNAs in the absence of Cas9 protein (Fig. 1A, quantified in Fig. 1B). To ensure STAG2 KO did not affect cell proliferation, and thus endpoint viability, over the course of any drug treatments, we assessed the proliferation of control and KO cell lines over 96 h. In line with previous literature in bladder cancer [12, 25], STAG2 KO did not significantly alter the degree of T24 cell proliferation (Fig. 1C). This suggests that although STAG2 acts in an oncogenic manner in MIBC, it is not critical for maintaining the proliferative capacity of MIBC cells.

To identify drugs that are differentially effective based on STAG2 expression, we screened a panel of 312 experimental and FDA-approved compounds with well characterized targets on each cell line (STAG2 WT: A6; STAG2 KO: G2 and H2) (Table S1). Cells were treated with the compound library at a concentration of 534 nM for 72 h then analyzed for cell viability via resazurin assay. The initial screening concentration was selected based on the technical capabilities of the robotic drug dispensing equipment and to minimize off-target cytotoxicity at higher doses. The distribution of viability of each cell line after library drug treatment is summarized in Fig. 1D. To select STAG2 differential drug hits, we calculated the difference in percent viability between STAG2 WT and KO cells for each compound and set a threshold to identify at least 10 compounds that more effectively target either control A6 cells or STAG2 KO cells. As shown in Fig. 1E, several drugs were identified as differentially effective based on STAG2 expression, with 71 drugs more cytotoxic in either of the STAG2 KO clonal cell lines compared to controls and 29 drugs specifically more cytotoxic to the control cell line (Table 1).

**Table 1:** STAG2 KO sensitizers and protectors.

Drug	T24 A6 (Control) Viability, %	T24 G2 (KO) Viability, %	T24 H2 (KO) Viability, %	G2 Viability - A6 Viability (%)	H2 Viability - A6 Viability (%)	Categorization
Ridaforolimus	91.48	91.26	70.93	−0.22	**−20.55**	STAG2 KO Sensitizer
Irinotecan	73.08	78.63	62.25	5.55	**−10.83**	STAG2 KO Sensitizer
Trichostatin A	82.60	82.77	66.50	0.18	**−16.10**	STAG2 KO Sensitizer
Rigosertib	32.73	20.71	12.10	**−12.02**	**−20.63**	STAG2 KO Sensitizer
PIK-93	91.37	99.58	80.72	8.21	**−10.65**	STAG2 KO Sensitizer
KRN 633	110.29	104.40	99.15	−5.89	**−11.14**	STAG2 KO Sensitizer
Degrasyn	108.16	101.21	94.55	−6.95	**−13.61**	STAG2 KO Sensitizer
KX2-391	31.75	21.10	16.91	**−10.65**	**−14.84**	STAG2 KO Sensitizer
Bosutinib	87.65	82.15	77.89	−5.50	**−9.76**	STAG2 KO Sensitizer
Vorinostat	85.33	68.27	87.93	**−17.07**	2.59	STAG2 KO Sensitizer
Ruxolitinib	94.34	78.24	93.11	**−16.09**	−1.23	STAG2 KO Sensitizer
TSU-68	97.68	91.06	79.00	−6.62	**−18.67**	STAG2 KO Sensitizer
MLN8054	79.47	68.91	86.04	**−10.55**	6.57	STAG2 KO Sensitizer
TAE684	80.06	56.97	69.66	**−23.09**	**−10.40**	STAG2 KO Sensitizer
BIX 02189	94.80	77.55	94.05	**−17.25**	−0.75	STAG2 KO Sensitizer
XL147 analogue	100.31	92.09	90.22	−8.22	**−10.09**	STAG2 KO Sensitizer
Ipatasertib	90.64	91.59	79.27	0.96	**−11.37**	STAG2 KO Sensitizer
CX-4945	91.08	82.62	78.29	−8.46	**−12.80**	STAG2 KO Sensitizer
A66	85.93	77.99	70.63	−7.94	**−15.29**	STAG2 KO Sensitizer
EPZ5676	90.97	92.13	73.83	1.17	**−17.14**	STAG2 KO Sensitizer
PF-00562271	79.52	69.93	70.15	**−9.59**	**−9.37**	STAG2 KO Sensitizer
Crenolanib	94.33	87.49	78.37	−6.85	**−15.96**	STAG2 KO Sensitizer
AZ 628	90.29	79.64	87.81	**−10.65**	−2.48	STAG2 KO Sensitizer
Regorafenib	95.87	86.84	95.32	**−9.03**	−0.55	STAG2 KO Sensitizer
BX-912	92.73	77.24	75.79	**−15.49**	**−16.94**	STAG2 KO Sensitizer
AZD7762	79.28	71.71	65.42	−7.57	**−13.87**	STAG2 KO Sensitizer
Cabozantinib	97.96	72.82	87.80	**−25.14**	**−10.16**	STAG2 KO Sensitizer
PD318088	70.13	56.91	65.82	**−13.22**	−4.32	STAG2 KO Sensitizer
GSK461364	37.66	19.70	17.36	**−17.96**	**−20.30**	STAG2 KO Sensitizer
Indirubin	89.53	83.37	76.67	−6.16	**−12.86**	STAG2 KO Sensitizer
Piceatannol	92.99	77.20	93.73	**−15.79**	0.74	STAG2 KO Sensitizer
GSK J4 HCl	92.75	72.53	91.59	**−20.22**	−1.16	STAG2 KO Sensitizer
Y-27632 2HCl	95.74	86.40	76.99	**−9.34**	**−18.75**	STAG2 KO Sensitizer
Brivanib	91.92	74.72	88.98	**−17.20**	−2.94	STAG2 KO Sensitizer
U0126-EtOH	86.16	85.01	66.65	−1.15	**−19.51**	STAG2 KO Sensitizer
Foretinib	86.76	78.96	73.34	−7.80	**−13.42**	STAG2 KO Sensitizer
R406	74.24	61.54	66.69	**−12.69**	−7.55	STAG2 KO Sensitizer
Everolimus	80.53	75.95	62.89	−4.58	**−17.65**	STAG2 KO Sensitizer
KU-60019	91.90	91.41	80.39	−0.50	**−11.51**	STAG2 KO Sensitizer
Alpelisib	88.12	74.41	77.06	**−13.71**	**−11.06**	STAG2 KO Sensitizer
SGC 0946	94.49	66.01	80.75	**−28.47**	**−13.74**	STAG2 KO Sensitizer
Flavopiridol	47.98	33.00	43.75	**−14.98**	−4.23	STAG2 KO Sensitizer
Resminostat	77.41	65.58	71.52	**−11.83**	−5.88	STAG2 KO Sensitizer
PKI-402	60.15	75.24	49.50	15.09	**−10.65**	STAG2 KO Sensitizer
Sunitinib Malate	87.87	83.56	70.80	−4.31	**−17.07**	STAG2 KO Sensitizer
TG100-115	90.52	61.59	83.66	**−28.93**	−6.86	STAG2 KO Sensitizer
ZSTK474	73.66	77.46	60.29	3.79	**−13.37**	STAG2 KO Sensitizer
HMN-214	50.59	35.61	46.45	**−14.98**	−4.14	STAG2 KO Sensitizer
ZM 447439	87.61	78.13	77.21	**−9.47**	**−10.40**	STAG2 KO Sensitizer
SGX-523	88.91	84.83	73.50	−4.08	**−15.40**	STAG2 KO Sensitizer
CP-673451	88.17	80.10	76.93	−8.06	**−11.24**	STAG2 KO Sensitizer
BS-181 HCl	95.44	81.65	89.67	**−13.79**	−5.77	STAG2 KO Sensitizer
Tyrphostin AG 879	97.34	83.36	85.16	**−13.98**	**−12.18**	STAG2 KO Sensitizer
WYE-125132	54.30	41.34	49.12	**−12.96**	−5.18	STAG2 KO Sensitizer
IWR-1-endo	92.47	74.50	70.23	**−17.97**	**−22.25**	STAG2 KO Sensitizer
Apitolisib	71.24	48.40	57.48	**−22.84**	**−13.76**	STAG2 KO Sensitizer
PD184352	78.82	62.21	68.40	**−16.61**	**−10.42**	STAG2 KO Sensitizer
Enzastaurin	73.46	52.14	57.49	**−21.33**	**−15.97**	STAG2 KO Sensitizer
GSK1059615	73.50	70.04	60.26	−3.46	**−13.24**	STAG2 KO Sensitizer
OSI-906	91.35	71.84	88.73	**−19.51**	−2.62	STAG2 KO Sensitizer
AEE788	91.62	82.55	81.98	**−9.08**	**−9.65**	STAG2 KO Sensitizer
GSK690693	98.38	86.89	94.21	**−11.49**	−4.18	STAG2 KO Sensitizer
Paclitaxel	20.26	9.36	11.09	**−10.90**	**−9.18**	STAG2 KO Sensitizer
WP1066	90.79	74.00	80.80	**−16.79**	**−9.99**	STAG2 KO Sensitizer
Volasertib	55.34	54.54	37.89	−0.80	**−17.45**	STAG2 KO Sensitizer
VX-702	93.15	84.03	97.17	**−9.12**	4.02	STAG2 KO Sensitizer
ICG-001	98.04	87.25	95.26	**−10.79**	−2.78	STAG2 KO Sensitizer
Tivantinib	72.10	49.25	72.04	**−22.85**	−0.06	STAG2 KO Sensitizer
BEZ235	70.67	60.35	54.63	**−10.32**	**−16.04**	STAG2 KO Sensitizer
Palomid 529	91.17	80.29	91.19	**−10.88**	0.02	STAG2 KO Sensitizer
PF-04691502	68.92	69.44	52.57	0.52	**−16.34**	STAG2 KO Sensitizer
Masitinib	96.91	106.49	100.28	**9.58**	3.37	STAG2 KO Protector
Pemetrexed	86.70	106.05	105.69	**19.35**	**18.98**	STAG2 KO Protector
NVP-BHG712	94.10	106.11	99.49	**12.00**	5.38	STAG2 KO Protector
TAK-733	70.86	79.99	80.41	**9.13**	**9.55**	STAG2 KO Protector
CHIR-124	78.60	83.76	89.55	5.16	**10.95**	STAG2 KO Protector
PD0325901	62.16	62.98	74.31	0.82	**12.15**	STAG2 KO Protector
Pictilisib	76.83	87.81	85.52	**10.98**	8.68	STAG2 KO Protector
Ponatinib	68.09	82.19	61.83	**14.11**	−6.25	STAG2 KO Protector
LDN-193189	81.48	93.38	82.53	**11.90**	1.05	STAG2 KO Protector
Tivozanib	84.76	88.41	94.54	3.64	**9.78**	STAG2 KO Protector
PI-103	50.54	74.06	78.62	**23.52**	**28.08**	STAG2 KO Protector
R788	79.61	90.35	81.33	**10.73**	1.72	STAG2 KO Protector
AZD5438	58.22	73.44	70.15	**15.21**	**11.92**	STAG2 KO Protector
WAY-600	73.37	78.66	83.75	5.28	**10.38**	STAG2 KO Protector
Rapamycin	59.06	81.16	67.46	**22.10**	8.40	STAG2 KO Protector
Pelitinib	50.54	68.77	60.12	**18.23**	**9.58**	STAG2 KO Protector
Pimasertib	58.26	51.09	68.23	−7.17	**9.97**	STAG2 KO Protector
SNS-032	96.01	106.59	106.96	**10.58**	**10.95**	STAG2 KO Protector
BMS-265246	66.23	88.81	70.08	**22.57**	3.85	STAG2 KO Protector
PP121	55.02	66.26	61.96	**11.24**	6.94	STAG2 KO Protector
Trametinib	54.56	53.74	64.22	−0.82	**9.66**	STAG2 KO Protector
Dovitinib	86.04	79.49	95.39	−6.55	**9.35**	STAG2 KO Protector
Sorafenib Tosylate	80.74	96.98	93.41	**16.24**	**12.67**	STAG2 KO Protector
Amuvatinib	82.89	76.30	95.38	−6.59	**12.49**	STAG2 KO Protector
OSI-027	57.49	58.22	68.54	0.72	**11.04**	STAG2 KO Protector
PKI-402	60.15	75.24	49.50	**15.09**	−10.65	STAG2 KO Protector
Roxadustat	95.80	104.86	96.12	**9.06**	0.33	STAG2 KO Protector
PLX-4720	91.75	105.83	97.75	**14.08**	6.00	STAG2 KO Protector
IMD 0354	80.03	90.21	83.51	**10.18**	3.47	STAG2 KO Protector

All drugs that were identified as hits in either G2/A6 or H2/A6 comparisons. Bold indicates the difference in viability that classified the drug as a hit. Drugs were classified as STAG2 KO sensitizers if the drug was more effective in the STAG2 KO (G2 or H2) cell lines and were classified as STAG2 KO protectors if they were more effective in the STAG2 WT (A6) cell line.

For all compounds meeting the differential sensitivity threshold, we quantified the number of shared molecular targets for compounds displaying efficacy in either STAG2 KO or WT cells, while also examining the overall representation of these targets in the total compound library. We grouped all drugs that were classified as ‘STAG2 KO Sensitizers,’ that is loss of STAG2 resulted in greater sensitivity to the drug, and for all hits classified as ‘STAG2 KO protectors,’ for which the drug was less effective in STAG2 KO cells and more effective in STAG2 WT cells. The targets most frequently represented in the group of STAG2 KO sensitizers were inhibitors of PI3K, PLK, mTOR, and MEK (Fig. 1F). The targets most frequently represented in the STAG2 KO protectors targeted MEK, CDK, mTOR and PI3K (Fig. 1G). It is noted that there was a modest overlap in the target classes between STAG2 sensitizers and STAG2 protectors. For example, 11 of the available PI3K inhibitors (specific compounds listed in Fig. 1F, including compounds targeting multiple molecules in addition to PI3K) screened were classified as STAG2 KO sensitizers, while three different PI3K-targeting compounds were classified as STAG2 KO protectors (specific compounds listed in Fig. 1G, including compounds targeting multiple molecules in addition to MEK). An additional 12 PI3K-targeting compounds screened showed no STAG2 differential efficacy. This suggests that drugs targeting the same pathway but with different mechanisms of action, secondary targets, or isoform selectivity (such as PI3K inhibitors with different selectivity for p110ɑ/β/γ/δ) may exert differential cytotoxicity depending on STAG2 expression.

MEK and PI3K were enriched targets in the STAG2 KO protector setting (Fig. 1F, G). To identify the specific drugs with the largest difference in efficacy between control cells and STAG2 KO cells, we investigated each individual MEK and PI3K inhibitor included in the drug screen (Fig. S1A and S1B). Of all drugs included in the compound screen, PI3K inhibitor PI-103 and the MEK inhibitor TAK-733 both exhibited the greatest cytotoxicity against control cell line A6 compared to STAG2 KO G2 and H2, and were therefore selected for further testing (Fig. 1H, left and middle) (Cell viability, PI-103: Control A6 50.54%, STAG2 KO G2 74.06%, STAG2 KO H2 78.62%; TAK-733: Control A6 70.86%, STAG2 KO G2 79.99%, STAG2 KO H2 80.41%). Conversely, drugs considered STAG2 KO sensitizers in at least one STAG2 KO cell line included approximately 80% of all available PLK inhibitors from the drug screen (Fig. S1C). The PLK1 inhibitor rigosertib showed increased efficacy against both STAG2 KO clones in the single dose screen (Cell viability, Control A6: 32.73%; STAG2 KO G2: 20.71%; STAG2 KO H2: 12.1%), and therefore we chose to validate this compound as a STAG2 KO sensitizer (Fig. 1H, right).

### In silico analysis of DepMap bladder cancer cell lines indicates that low STAG2 mRNA expression is associated with increased sensitivity to CHK and ATR inhibition

**Fig. 2:**
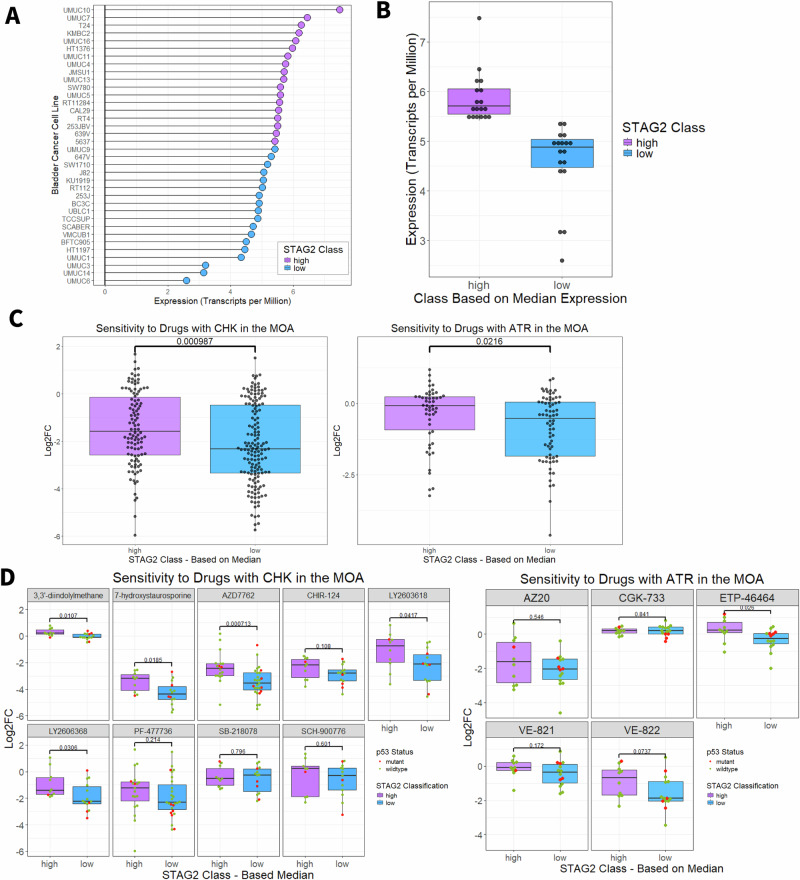
In silico analysis of DepMap bladder cancer cell lines indicates that low STAG2 mRNA expression drives sensitivity to CHK and ATR inhibition. **A** Lollipop plot indicating the STAG2 mRNA expression in transcripts per million of each individual bladder cancer cell line from the DepMap database. Blue indicates cell lines classified as STAG2 low (expression below median, *n* = 18) and purple indicates cell lines classified as STAG2 high (expression above median, *n* = 18). **B** STAG2 mRNA expression in transcripts per million for STAG2 high and STAG2 low groups of bladder cancer cell lines from the DepMap database. Boxplots indicate first quartile, median, and third quartile. **C** Log2FC relative to DMSO controls of STAG2 high and STAG2 low DepMap bladder cancer cell lines after treatment with drugs that include CHK (left), or ATR (right) listed in their mechanism of action. An increasingly negative Log2FC indicates increasing sensitivity. -values calculated with two sample Wilcoxon test. **D** Log2FC of STAG2 high and STAG2 low DepMap bladder cancer cell lines after treatment with individual inhibitors targeting CHK (left) or ATR (right). Red indicates cell lines that are p53 mutant, green indicates cell lines that are p53 wildtype. *P*-values calculated with two sample Wilcoxon test. *p < 0.05; ***p < 0.001. MOA Mechanism of action, Log2FC Log2 fold change, CHK checkpoint kinase, ATR ataxia telangiectasia and Rad3-related. Boxplots indicate first quartile, median, and third quartile.

To examine additional drugs that may have differential effects on bladder cancer cell lines based on STAG2 expression, we queried the PRISM dataset consisting of cell viability data from pooled-cell line chemical perturbation screens of over 4500 compounds and available through the Dependency Map (DepMap) portal [26]. To begin, we examined the mRNA expression of STAG2 in each cell line (Fig. 2A). Cell lines were classified as STAG2 high or STAG2 low based on the median expression of the total collection of bladder cancer cell lines (Fig. 2B). Following this, comparisons were made between groups to identify drugs that were significantly more effective in STAG2 high or STAG2 low expressing bladder cancer cell lines. Cell lines classified as STAG2 low were significantly more sensitive to drugs targeting CHK (Fig. 2C, left) and ATR (Fig. 2C, right) measured as the log_2_ fold change relative to DMSO treated controls. This is in support of previously published work which implicates STAG2 in the DNA damage response pathway, along with both ATR and CHK [2–4].

To further delineate which specific CHK- and ATR-targeting compounds were most effective against STAG2-low cell lines, we individually assessed each CHK and ART inhibitor in the DepMap database across the STAG2 expression classes. Two CHK inhibitors had the largest difference in efficacy between the STAG2 low and STAG2 high cell lines – LY2603618 (median LFC, STAG2 low: −2.13, STAG2 high: −0.762; *p* = 0.0417, Wilcoxon rank sum test) and LY2606368 (median LFC, STAG2 low: −2.23, STAG2 high: −1.42; *p* = 0.0306, Wilcoxon rank sum test) (Fig. 2D, left). Although we identified LY2603618 as a promising agent in our study, this drug did not show efficacy in clinical trials. Despite a favorable safety profile, LY2603618 failed to improve overall survival, progression-free survival, or duration of response in a phase II study of pancreatic cancer patients [27]. Conversely, LY2606368 has demonstrated durable single-agent activity in ovarian cancer [28]. Although both compounds appear similarly effective in bladder cancer, we selected LY2606368, also known as prexasertib, as a candidate CHK inhibitor to pursue for further testing. From the DepMap database, there are five compounds targeting ATR (Fig. 2D, right). The compound VE-822 was more effective against STAG2 low compared to STAG2 high cell lines (median log fold change (LFC), STAG2 low: −1.87, STAG2 high: −0.689; difference: 1.181). VE-822, also known as berzosertib, VX970, or M6620, is a highly selective ATR inhibitor which diminishes ATR-CHK1 signaling in response to DNA damage. As p53 is a key player in cell cycle arrest in response to DNA damage, we assessed the contribution of p53 mutational status to ATR and CHK inhibitor sensitivity. As shown in Fig. 2D, p53 mutations occur more frequently in the STAG2 low group (*n* = 4 p53 mutant cell lines) compared to the STAG2 high group (*n* = 1 p53 mutant cell line); however, there were no significant differences in sensitivity to any CHK or ATR inhibitor except for CGK-733 (*p* = 0.002) when comparing p53 mutant and wild-type cell lines within the STAG2 low expression class (Wilcoxon rank sum test, *p* > 0.05) (Fig. S2B). Therefore, we conclude that the differential cytotoxicity of prexasertib and berzosertib between the STAG2 low and high groups is not influenced by p53 mutation status. We have previously shown that knockdown of STAG2 does not affect cell cycle distribution in MIBC cells [12]. Therefore, the observed differences in sensitivity to CHK or ATR inhibition are not necessarily due to different levels of STAG2 inducing accumulation in one phase of the cell cycle.

Together with our in vitro drug screen results, we identified two inhibitors to investigate as STAG2 KO protectors (PI-103 and TAK-733) and three inhibitors to investigate as STAG2 KO sensitizers (Rigosertib, Prexasertib, and Berzosertib). Additionally, we asked whether the drugs identified from the screen (TAK-733, PI-103, and Rigosertib) showed similar patterns of efficacy against DepMap bladder cancer cell lines. The average Log2FC in viability of STAG2 low cell lines relative to DMSO controls was lower upon rigosertib treatment and higher after both PI-103 and TAK-733 treatment compared to the group of STAG2 high cell lines (Fig. S2A), which lends support to screening results which identified rigosertib as a STAG2 KO sensitizer and PI-103 and TAK-733 as STAG2 KO protectors.

A growing body of literature suggests that STAG2 deletion or loss-of-function truncating mutations sensitize cancer cells to PARP inhibitors, including olaparib, rucaparib, and veliparib, although to date these studies have not been conducted in bladder cancer [2, 16, 17]. Bladder cancer cell lines with available data in DepMap do not exhibit differential sensitivity to inhibitors targeting PARP overall (Fig. S3A) or to any individual PARP inhibitor aside from the second-generation PARP inhibitor talazoparib (Fig. S3B). It is established that mutations in the BRCA1 or BRCA2 gene underlie sensitivity to PARP inhibitors in cancer cells, most notably in breast cancer [29]. All the examined bladder cancer cell lines were BRCA1 and BRCA2 wild-type, and therefore the observed viability is not influenced by BRCA mutational status. Although there were minimal differences in sensitivity based on STAG2 status, DepMap drug sensitivity data is limited to a single treatment dose (2.5 µM), and therefore may not fully capture differences in sensitivity that occur at different doses. Therefore, we chose to include the PARP inhibitors olaparib and talazoparib in validation testing over a wide range of doses.

Along with MEK, PI3K, and PLK1 inhibitors, our list of candidate drugs included ATR, CHK, and PARP inhibitors that are all important members of the DNA damage response pathway. Previously, we identified that loss of STAG2 protein expression by shRNA knockdown leads to profound transcriptional changes in T24 cells as measured by RNA-sequencing [12]. We queried these data to ask whether loss of STAG2 protein expression alters enrichment of pathways related to these investigated drug targets. We found that pathways related to DNA damage response are enriched in the STAG2 KD setting (Fig. S3C). The integration of transcriptomic and drug sensitivity data suggests that loss of functional STAG2 drives dependency on the DNA damage response pathway, and, therefore, increases sensitivity to ATR, CHK, and PARP inhibitors. PI3K signaling, regulation of PI3K signaling, and regulation of MAPK cascade signaling pathways were enriched in the control (STAG2 WT) setting (Fig. S3C). This further suggests that targeting MEK and PI3K may be more effective against STAG2 WT compared to cells with reduced expression or functional loss of STAG2, which corroborates our drug screening data. Altogether, changes in the transcriptome after altering STAG2 expression fully support the list of candidate drugs identified from our custom drug screen, DepMap analysis, and drug sensitivities reported in the literature [2, 16, 17].

### Loss of STAG2 protein expression increases sensitivity to berzosertib and olaparib and decreases sensitivity to PI-103 in T24 cells

**Fig. 3:**
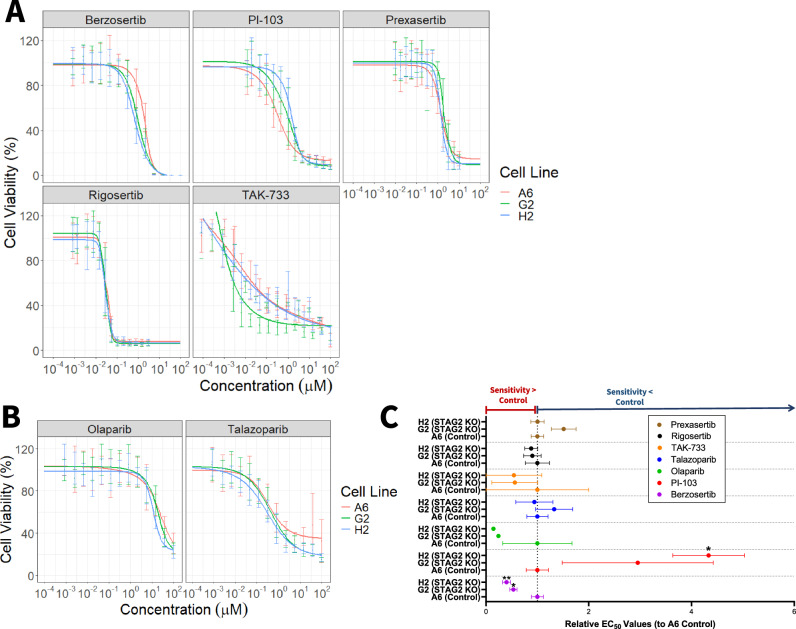
Loss of STAG2 protein expression increases sensitivity to berzosertib and olaparib and decreases sensitivity to PI-103 in T24 cells. **A** Dose response curves for T24 control A6 (red), T24 STAG2 KO G2 (green), and T24 STAG2 KO H2 (blue) cell viability after treatment with the indicated candidate drugs and **B** PARP inhibitors olaparib and talazoparib at doses ranging from 10^-4 ^µM to 10^2 ^µM for 72 h. Curves modeled using package drda in R; representative of at least three independent experiments. Error bars represent standard deviation. Cell viability normalized and calculated as a percentage compared to untreated wells within each individual experiment. **C** Relative EC_50_ values for T24 control A6, STAG2 KO G2, and STAG2 KO H2 cell lines derived from three independent experiments as displayed in (**A**, **B**). A relative EC_50_ value greater than one indicates that the cell line has a higher EC_50_ than control and therefore is less sensitive to the drug. A relative EC_50_ value lower than one indicates that the cell line has a lower EC_50_ than control, and therefore is more sensitive to the drug. Statistical significance analyzed via t-test; *p < 0.05.

To test whether sensitivity to our panel of candidate drugs (PI-103, TAK-733, rigosertib, prexasertib, berzosertib) and chosen PARP inhibitors (olaparib and talazoparib) significantly differed based on STAG2 expression level, we performed dose response testing on STAG2 KO cell lines G2 and H2 and control cell line A6. Cells were seeded at 500 cells per well to ensure that they did not reach 100% confluency by the end of the experiment, then were treated with candidate drugs for 72 h. We modeled 4-parameter log-logistic dose response curves for each cell line-drug combination tested and calculated half maximal effective concentrations (EC_50_). Dose-response curves for the panel of candidate drugs are shown in Fig. 3A. We found that relative to T24 Control A6 cells, STAG2 KO sensitized cells to the ATR inhibitor berzosertib (Relative EC_50_ values - Control A6: 1.00; STAG2 KO G2: 0.53; STAG2 KO H2: 0.40), which supports our hypothesis that STAG2 deficient cells are more sensitive to drugs targeting DNA damage response proteins (Fig. 3A, C, Table 2). Additionally, T24 cells with intact STAG2 expression are more sensitive to treatment with PI3K inhibitor PI-103 (Relative EC_50_ values - Control A6: 1.00; STAG2 KO G: 22.95; STAG2 KO H2: 4.33) (Fig. 3A, Table 2). We did not identify any difference in efficacy based on STAG2 status (control versus KO) for prexasertib, rigosertib, or TAK-733 based on EC_50_ values (Table 2).

**Table 2:** T24 EC50 values.

		EC_50_ (µM)	
Drug	Cell Line	Mean	SEM
Berzosertib	Control A6	1.936	0.2246
	STAG2 KO G2	1.033	0.1367
	STAG2 KO H2	0.7738	0.1474
PI-103	Control A6	0.3269	0.07084
	STAG2 KO G2	0.9653	0.4807
	STAG2 KO H2	1.416	0.2284
Prexasertib	Control A6	1.443	0.1758
	STAG2 KO G2	2.182	0.3476
	STAG2 KO H2	1.446	0.1872
Rigosertib	Control A6	0.03457	0.008127
	STAG2 KO G2	0.03118	0.005892
	STAG2 KO H2	0.03039	0.004357
TAK-733	Control A6	0.003291	0.003291
	STAG2 KO G2	0.001842	0.001461
	STAG2 KO H2	0.001788	0.001788
Olaparib	Control A6	79.9	53.83
	STAG2 KO G2	19.3	2.326
	STAG2 KO H2	11.52	0.8954
Talazoparib	Control A6	0.3487	0.07194
	STAG2 KO G2	0.4626	0.126
	STAG2 KO H2	0.3282	0.1258

Average and SEM of EC_50_ values (µM) calculated from dose response curves for each cell line and drug combination tested in T24 cells. Each EC_50_ is representative of three independent experiments.

T24 cells were not differentially sensitive to talazoparib or olaparib based on STAG2 status, however they were significantly more sensitive to talazoparib compared to olaparib overall as evidenced by much lower EC_50_ values (Control A6: 0.3487 µM vs 79.90 µM; STAG2 KO G2 0.4626 µM vs 19.30 µM; STAG2 KO H2 0.3282 µM vs 11.52 µM (Fig. 3B). Although we did not identify differences in sensitivity based on STAG2 protein expression, the general effectiveness of talazoparib against T24 cells may warrant further investigation as a therapy for MIBC.

To extend our findings to another in vitro KO model system, we utilized CRISPR-Cas9 to KO STAG2 expression in TCCSUP MIBC cells that have high endogenous STAG2 expression. We quantified STAG2 expression in two TCCSUP non-targeting control cell lines (CC1 and CC2) and two STAG2 KO clones (CS2-2 and CS2-3) via western blot (Fig. S4A). Similar to our T24 model system, STAG2 KO did not impact cell proliferation over the course of 96 h (Fig. S4B); however, these cells proliferate at a much slower rate than T24 cells (between 300% and 600% increase in cell number for TCCSUP compared to 1200–1500% for T24 cells after 96 h). We observed no significant differences in sensitivity to our compound panel based on STAG2 expression, except for STAG2 CS2-3 (KO-3), which was significantly less sensitive to berzosertib treatment compared to the control cell lines. (Fig. S4C, S4D, and Table S2). Since many of these drugs target rapidly proliferating cells, TCCSUP cells may be less sensitive to them in general due to their slow proliferation rate. This may mask differences based on STAG2 expression alone. Future studies that evaluate longer term drug treatments would determine whether this plays a major role in sensitivity to these drugs.

Finally, we sought to test candidate drugs in an shRNA-mediated STAG2 knockdown (KD) system. While shRNA-mediated knockdown (KD) does not result in complete knockout, it minimizes differences caused by clonal selection that can arise when generating CRISPR KO cell lines. We utilized the well-established HB-CLS-1 cell line and a patient derived bladder cancer cell line, BO1, which both have endogenous expression of STAG2 (Fig. S5A). shRNA-mediated KD successfully reduced STAG2 protein expression in both cell lines (Fig. S5A). Overall, the dose-response of BO1 cell lines was consistent with the T24 data. BO1-shSTAG2 cells showed reduced sensitivity to PI-103 and TAK-733 compared to controls, however, the differences did not reach statistical significance due to high variability between experiments (Fig. S5B, S5C). BO1-shSTAG2 cells showed significantly increased sensitivity to talazoparib and a slight, though nonsignificant, trend toward higher sensitivity to olaparib, consistent with literature indicating that STAG2 loss enhances sensitivity to PARP inhibitors. (Fig. S5B, S5C). Dose-response of HB-CLS-1 cell lines was highly variable, making statistical analysis challenging (Fig. S5D, S5E); however, HB-CLS-1 shSTAG2 cells were highly sensitive to olaparib, which is consistent with previous results.

### Berzosertib and cisplatin exhibit synergistic cytotoxicity in MIBC cells

**Fig. 4:**
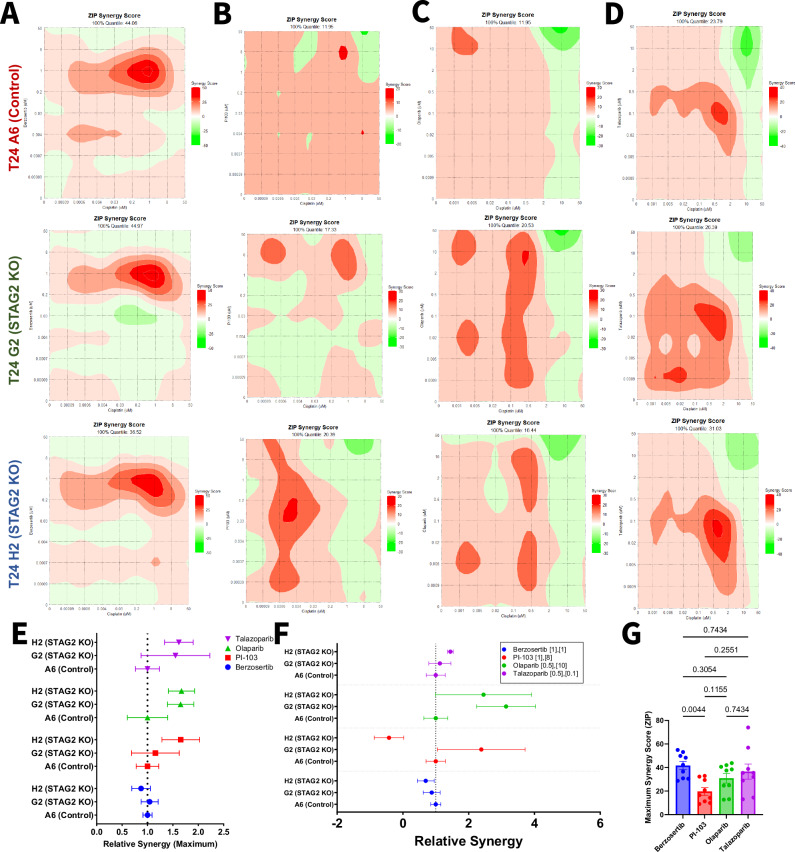
Berzosertib, olaparib, and talazoparib exhibit synergy in combination with cisplatin in MIBC cells. Contour plots representing ZIP synergy scores for the combination of **A** berzosertib, **B** PI-103, **C** Olaparib, **D** or talazoparib with cisplatin calculated using the ZIP synergy model for T24 control A6 (left), T24 STAG2 KO G2 (middle), and T24 STAG2 KO H2 (right). 100% quantile represents the maximum synergy score achieved in each cell line for the combination. **E** Ratio of the maximum synergy score for each cell line relative to T24 Control A6. A maximum synergy ratio greater than one indicates that the drug combination was more synergistic in the indicated cell line compared to control. A maximum synergy ratio less than one indicates that the drug combination was less synergistic in the indicated cell line compared to control. **F** Ratio of the synergy score for each cell line relative to T24 Control A6 at the specified concentrations (µM) of cisplatin and indicated candidate drug. A synergy ratio greater than one indicates that the drug combination was more synergistic in the indicated cell line compared to control. A synergy ratio less than one indicates that the drug combination was less synergistic in the indicated cell line compared to control. **G** Maximum ZIP synergy scores of indicated drugs in combination with cisplatin in all T24 cell lines combined. Statistical differences analyzed via one-way ANOVA with multiple comparisons.

Dose-response studies revealed that the ATR inhibitor berzosertib and the PARP inhibitor talazoparib are more cytotoxic to STAG2 low and KO cells, while the PI3K inhibitor PI-103 is more cytotoxic in the STAG2 high context. To examine whether these drugs may have translational potential, we sought to identify whether these drugs exhibit synergism with cisplatin against bladder cancer cells. For comparison to talazoparib, we also tested the combination of cisplatin and olaparib. We treated STAG2 WT (control A6) and KO (G2 and H2) clones with cisplatin in combination with berzosertib, PI-103, olaparib, and talazoparib in increasing concentrations ranging from 0.0009 to 50 µM. Dose response combination matrices for each drug combination in each cell line can be found in Fig. S6. By the ZIP synergy modeling method, all cell lines tested exhibited significant synergy between cisplatin and berzosertib, most notably at 1 µM concentrations of each compound (Fig. 4A). Synergy scores for the combination of cisplatin and PI-103 were smaller in magnitude and more variable across drug concentrations and cell lines (Fig. 4B) Finally, we assessed synergism between the individual PARP inhibitors, olaparib and talazoparib, in combination with cisplatin. Both olaparib and talazoparib achieved maximum synergy scores over 20 in all three cell lines tested (Fig. 1C, D). To analyze whether synergy depends on STAG2 status, we calculated the maximum synergy ratio for each cell line (maximum synergy score divided by maximum synergy score of the control). A higher synergy ratio indicates greater synergy in the tested cell line compared to the control, while a lower synergy ratio suggests reduced synergy relative to the control (Fig. 4D). There were no significant differences in synergy ratios between the STAG2 KO G2 and H2 cell lines compared to control A6, implying that the synergy between the tested compounds and cisplatin is not necessarily STAG2 dependent (Fig. 4E). We further analyzed synergy ratios at specific doses of each drug combination based on maximum synergy doses of three independent experiments (Fig. 4F), but did not identify any significant differences. As there were no differences based on STAG2 status, we then pooled the maximum synergy score for all cell lines within each drug combination (Fig. 4G). This data suggests that berzosertib is significantly more synergistic than PI-103 in combination with cisplatin (Fig. 4G).

We conducted synergy testing in the BO1 model system to further validate these findings (Figs. S7 and S8). In line with our T24 model system, we did not identify any STAG2-specific differences in relative maximum synergy (Fig. S8E). We then pooled the maximum synergy score for all cell lines within the drug combinations and found that berzosertib is significantly more synergistic than PI-103, olaparib, or talazoparib in combination with cisplatin (Fig. 4G). These results provide rationale for further investigation of berzosertib in combination with cisplatin as novel therapeutic approach for MIBC patients.

## Discussion

While STAG2 is categorized as a tumor suppressor in many cancer types, its role is tissue specific. Previous work from our group and others supports the notion that STAG2 acts in an oncogenic manner in bladder cancer [9–12, 30]. Therefore, our overall objective for the current study was to identify therapeutic vulnerabilities based on STAG2 expression in bladder cancer cells.

Results from our drug screen demonstrate that STAG2 KO sensitized cells to 71 drugs and protected from 29 drugs. These drugs targeted a wide range of molecules, including but not limited to PI3K, MEK, PLK, and CDKs. Several PI3K inhibitors were more toxic for STAG2 expressing cells, while a separate group of PI3K inhibitors (which did not overlap with those selectively targeting STAG2 expressing cells) were more effective against STAG2 KO cells. In addition, some PI3K inhibitors did not exhibit any differential efficacy against MIBC cells based on STG2 expression. This range of response may be in part due to the individual drugs targeting different p110 catalytic subunits of the PI3K complex. Different p110 isoforms allow for fine-tuning of PI3K signaling and signal integration. For example, p110β has the unique capability of integrating stimuli from both receptor tyrosine kinases and G-protein coupled receptors (GPCRs) [31]. Analysis of transcriptional changes after STAG2 KD indicates that STAG2 may in fact play a role in GPCR signaling (Fig. S3C). It is possible that STAG2 KO decreases GPCR signaling activity, causing cells to be less reliant on p110β, and consequently altering sensitivity to inhibition of this specific p110 isoform.

We show that the pan-PI3K inhibitor PI-103 is more cytotoxic to T24 STAG2 WT cells, demonstrating a novel therapeutic approach in the STAG2-high setting for which there are no STAG2-specific therapies available to date. In a clinical setting, there is limited data detailing use of PI3K inhibitors in bladder cancer treatment. There has been a single phase 2 trial which investigated the pan-PI3K inhibitor, buparlisib, in metastatic urothelial carcinoma patients [32]. Unfortunately, the study was halted due to limited efficacy and treatment-related toxicities. However, the response appeared to correlate with mutations in the *TSC1* gene. This suggests that PI3K inhibition may be efficacious in a selected group of patients, such as those with high STAG2 expression.

We find that T24 STAG2 KO cells, and modestly T24 shSTAG2 cells, are more sensitive to ATR inhibition, specifically to treatment with the ATR inhibitor berzosertib. Early studies indicate that the addition of berzosertib to standard-of-care therapies is promising in lung cancer and advanced pancreatic, breast, head and neck, and colorectal cancers [33, 34]. One study has investigated berzosertib as a treatment for bladder cancer, where it was tested in a Phase 2 clinical trial in combination with cisplatin and gemcitabine. The trial ultimately did not reach its goal of increased PFS but did not use any genetic or biomarker-based patient selection. We posit that patients with STAG2 low or negative tumors may respond best to treatment with berzosertib, which may lead to successful benefits in clinical outcomes for this subset of patients.

Here, we demonstrate that berzosertib, olaparib, and talazoparib exhibit synergism when combined with cisplatin treatment. It is well established that berzosertib, olaparib, and talazoparib all play an important role in sensing and repair of DNA damage. STAG2 and the cohesin complex have a well-established role in DNA damage repair through homologous recombination [2–4]. Therefore, inducing DNA damage through cisplatin treatment and further inhibiting the repair of DNA damage through inhibition PARP is logically more effective in the STAG2 deficient setting. Further analysis of DNA damage within MIBC cells after combination treatment will identify how the loss of STAG2 may further hinder the DNA damage response in MIBC cells. We also found that berzosertib is the most significantly synergistic drug in combination with cisplatin across cell lines. As shown in dose-response data, berzosertib is the only drug capable of completely eliminating cell viability, whereas the efficacy of the other candidate drugs plateaus at a non-zero viability percentage. This may explain why berzosertib in particular is most capable of synergizing with cisplatin.

Our data demonstrates that inhibition of the PI3K pathway is more effective in the STAG2 WT setting whereas inhibition of ATR is more effective in the STAG2 KO setting. Our ATR results are in line with what would be expected given the role of STAG2 in the DNA damage response pathway. However, there is little known regarding the connection between STAG2 and the PI3K pathway. Based on our results showing that STAG2 WT cells are more sensitive to pan-PI3K inhibition, we speculate that this signaling pathway may be STAG2-regulated. In addition, our gene set enrichment analysis demonstrates that the status of STAG2 affects PI3K signaling components at the transcriptional level. It is established that STAG2 acts in a pro-tumorigenic manner in bladder cancer [9, 12, 35], and therefore STAG2 may exert an oncogenic function through regulation of PI3K signaling in bladder cancer cells. This may explain our findings in which T24 STAG2 WT cells were more sensitive to the PI3K inhibitor PI-103. This further is supported by in vitro work in osteosarcoma U2-OS cells, where loss of STAG2 is associated with decreased activation of the PI3K pathway, evidenced by decreased phosphorylation of PI3K, Akt, and mTOR [36]. Additional mechanistic studies identifying the direct role of STAG2 in regulating PI3K-associated genes will be necessary to further examine this relationship.

A strength of our study includes the use of a custom screening panel of compounds in addition to analysis of publicly available sensitivity data for over 4500 drugs through the DepMap portal. A limitation of these datasets is that they were performed at a single dose. It is possible that compounds with STAG2 differential sensitivity may not have been identified simply because the observed differential effect occurs at a dose higher or lower than what was captured by these two single-dose datasets. To address this, we took a comprehensive approach in analyzing our drug screen hits to identify whether there were any specific proteins that were frequently targeted, instead of considering drugs individually. This allowed us to expand our analysis to detect which shared targets were important in either the STAG2 intact or STAG2 KO setting.

Altogether, our study presents compelling evidence that berzosertib and PI-103 may be novel opportunities to investigate as precision medicine approaches for MIBC patients based on STAG2 tumor expression. Further, we show that berzosertib and cisplatin exhibit synergistic cytotoxicity against MIBC cells, a relationship which warrants further preclinical testing with models of varying STAG2 expression and mutational status. Finally, our results suggests that there is an unexplored connection between the role of STAG2 in gene regulation and the PI3K signaling axis, investigation of which may lead to other additional therapeutic strategies.

## Supplementary information

Supplemental Figures S1-S8, Tables S1 and S2, and Associated Legends

## Data Availability

All data generated during this study are included in this published article and its supplementary information files.

## Code availability

Code available upon request without restriction.

## References

[1] van der Lelij P, Lieb S, Jude J, Wutz G, Santos CP, Falkenberg K, et al. Synthetic lethality between the cohesin subunits STAG1 and STAG2 in diverse cancer contexts. eLife. 2017;6:e26980. 28691904 10.7554/eLife.26980 PMC5531830

[2] Tothova Z, Valton AL, Gorelov RA, Vallurupalli M, Krill-Burger JM, Holmes A, et al. Cohesin mutations alter DNA damage repair and chromatin structure and create therapeutic vulnerabilities in MDS/AML. JCI Insight. 2021;6(3):e142149. 33351783 10.1172/jci.insight.142149 PMC7934867

[3] Zhou J, Nie RC, He ZP, Cai XX, Chen JW, Lin WP, et al. STAG2 regulates homologous recombination repair and sensitivity to ATM inhibition. Adv Sci (Weinh). 2023;10(36):e2302494. 37985839 10.1002/advs.202302494 PMC10754142

[4] Kong X, Ball AR Jr, Pham HX, Zeng W, Chen H-Y, Schmiesing JA, et al. Distinct functions of human cohesin-SA1 and cohesin-SA2 in double-strand break repair. Mol Cell Biol. 2014;34(4):685–98. 24324008 10.1128/MCB.01503-13 PMC3911484

[5] Cuadrado A, Giménez-Llorente D, Kojic A, Rodríguez-Corsino M, Cuartero Y, Martín-Serrano G, et al. Specific contributions of Cohesin-SA1 and Cohesin-SA2 to TADs and polycomb domains in embryonic stem cells. Cell Rep. 2019;27(12):3500–10.e4. 31216471 10.1016/j.celrep.2019.05.078 PMC7057268

[6] Kojic A, Cuadrado A, De Koninck M, Giménez-Llorente D, Rodríguez-Corsino M, Gómez-López G, et al. Distinct roles of cohesin-SA1 and cohesin-SA2 in 3D chromosome organization. Nat Struct Mol Biol. 2018;25(6):496–504. 29867216 10.1038/s41594-018-0070-4 PMC6122591

[7] Richart L, Lapi E, Pancaldi V, Cuenca-Ardura M, Pau Enrique C-D-S, et al. STAG2 loss-of-function affects short-range genomic contacts and modulates the basal-luminal transcriptional program of bladder cancer cells. Nucleic Acids Res. 2021;49(19):11005–21. 34648034 10.1093/nar/gkab864 PMC8565347

[8] Casa V, Moronta Gines M, Gade Gusmao E, Slotman JA, Zirkel A, Josipovic N, et al. Redundant and specific roles of cohesin STAG subunits in chromatin looping and transcriptional control. Genome Res. 2020;30(4):515–27. 32253279 10.1101/gr.253211.119 PMC7197483

[9] Lelo A, Prip F, Harris BT, Solomon D, Berry DL, Chaldekas K, et al. STAG2 is a biomarker for prediction of recurrence and progression in papillary non-muscle-invasive bladder cancer. Clin Cancer Res. 2018;24(17):4145–53. 29954776 10.1158/1078-0432.CCR-17-3244 PMC6125225

[10] Taber A, Park Y, Lelo A, Prip F, Xiao J, Berry DL, et al. STAG2 as a prognostic biomarker in low-grade non-muscle invasive bladder cancer. Urol Oncol Semin Original Investig. 2021;39(7):438.e1–e9. 10.1016/j.urolonc.2021.02.007 PMC8286298 33712344

[11] Balbás-Martínez C, Sagrera A, Carrillo-de-Santa-Pau E, Earl J, Márquez M, Vazquez M, et al. Recurrent inactivation of STAG2 in bladder cancer is not associated with aneuploidy. Nat Genet. 2013;45(12):1464–9. 24121791 10.1038/ng.2799 PMC3840052

[12] Athans S, Krishnan N, Ramakrishnan S, Cortes Gomez E, Lage-Vickers S, Rak M, et al. STAG2 expression is associated with adverse survival outcomes and regulates cell phenotype in muscle-invasive bladder cancer. Cancer Res Commun. 2022;2(10):1129–43. 36275363 10.1158/2767-9764.CRC-22-0155 PMC9583756

[13] Evers L, Perez-Mancera PA, Lenkiewicz E, Tang N, Aust D, Knösel T, et al. STAG2 is a clinically relevant tumor suppressor in pancreatic ductal adenocarcinoma. Genome Med. 2014;6(1):9. 24484537 10.1186/gm526 PMC3971348

[14] Adane B, Alexe G, Seong BKA, Lu D, Hwang EE, Hnisz D, et al. STAG2 loss rewires oncogenic and developmental programs to promote metastasis in Ewing sarcoma. Cancer cell. 2021;39(6):827–44.e10. 34129824 10.1016/j.ccell.2021.05.007 PMC8378827

[15] Surdez D, Zaidi S, Grossetête S, Laud-Duval K, Ferre AS, Mous L, et al. STAG2 mutations alter CTCF-anchored loop extrusion, reduce cis-regulatory interactions and EWSR1-FLI1 activity in Ewing sarcoma. Cancer Cell. 2021;39(6):810–26.e9. 33930311 10.1016/j.ccell.2021.04.001

[16] Bailey ML, O’Neil NJ, van Pel DM, Solomon DA, Waldman T, Hieter P. Glioblastoma cells containing mutations in the cohesin component STAG2 are sensitive to PARP inhibition. Mol Cancer Ther. 2014;13(3):724–32. 24356817 10.1158/1535-7163.MCT-13-0749 PMC4130349

[17] Mondal G, Stevers M, Goode B, Ashworth A, Solomon DA. A requirement for STAG2 in replication fork progression creates a targetable synthetic lethality in cohesin-mutant cancers. Nat Commun. 2019;10(1):1686. 30975996 10.1038/s41467-019-09659-z PMC6459917

[18] Kimura S, Park CS, Montefiori LE, Iacobucci I, Polonen P, Gao Q, et al. Biologic and clinical analysis of childhood gamma delta T-ALL identifies LMO2/STAG2 rearrangements as extremely high-risk. Cancer Discov. 2024;14:1838–59. 38916500 10.1158/2159-8290.CD-23-1452 PMC11452281

[19] Wei L, Chintala S, Ciamporcero E, Ramakrishnan S, Elbanna M, Wang J, et al. Genomic profiling is predictive of response to cisplatin treatment but not to PI3K inhibition in bladder cancer patient-derived xenografts. Oncotarget. 2016;7(47):76374–89. 27823983 10.18632/oncotarget.13062 PMC5363516

[20] Vichai V, Kirtikara K. Sulforhodamine B colorimetric assay for cytotoxicity screening. Nat Protocols. 2006;1(3):1112–6. 17406391 10.1038/nprot.2006.179

[21] Kassambara A. rstatix: Pipe-Friendly Framework for Basic Statistical Tests. R package version 0.7.2. 2023. https://rpkgs.datanovia.com/rstatix/.

[22] Malyutina A, Tang J, Pessia A. drda: an R Package for dose-response data analysis using logistic functions. J Stat Softw. 2023;106(4):1–26.37138589

[23] Zheng S, Wang W, Aldahdooh J, Malyutina A, Shadbahr T, Tanoli Z, et al. SynergyFinder Plus: toward better interpretation and annotation of drug combination screening datasets. Genom Proteom Bioinform. 2022;20(3):587–96. 10.1016/j.gpb.2022.01.004 PMC9801064 35085776

[24] Tate JG, Bamford S, Jubb HC, Sondka Z, Beare DM, Bindal N, et al. COSMIC: the catalogue of somatic mutations in cancer. Nucleic Acids Res. 2018;47(D1):D941–D7. 10.1093/nar/gky1015 PMC6323903 30371878

[25] Solomon DA, Kim JS, Bondaruk J, Shariat SF, Wang ZF, Elkahloun AG, et al. Frequent truncating mutations of STAG2 in bladder cancer. Nat Genet. 2013;45(12):1428–30. 24121789 10.1038/ng.2800 PMC3875130

[26] Corsello SM, Nagari RT, Spangler RD, Rossen J, Kocak M, Bryan JG, et al. Discovering the anticancer potential of non-oncology drugs by systematic viability profiling. Nat Cancer. 2020;1(2):235–48. 32613204 10.1038/s43018-019-0018-6 PMC7328899

[27] Laquente B, Lopez-Martin J, Richards D, Illerhaus G, Chang DZ, Kim G, et al. A phase II study to evaluate LY2603618 in combination with gemcitabine in pancreatic cancer patients. BMC Cancer. 2017;17(1):137. 28202004 10.1186/s12885-017-3131-x PMC5312529

[28] Konstantinopoulos PA, Lee JM, Gao B, Miller R, Lee JY, Colombo N, et al. A phase 2 study of prexasertib (LY2606368) in platinum resistant or refractory recurrent ovarian cancer. Gynecol Oncol. 2022;167(2):213–25. 36192237 10.1016/j.ygyno.2022.09.019 PMC10673677

[29] Golan T, Hammel P, Reni M, Cutsem EV, Macarulla T, Hall MJ, et al. Maintenance Olaparib for Germline BRCA-mutated metastatic pancreatic cancer. N Engl J Med. 2019;381(4):317–27. 31157963 10.1056/NEJMoa1903387 PMC6810605

[30] Taylor CF, Platt FM, Hurst CD, Thygesen HH, Knowles MA. Frequent inactivating mutations of STAG2 in bladder cancer are associated with low tumour grade and stage and inversely related to chromosomal copy number changes. Hum Mol Genet. 2014;23(8):1964–74. 24270882 10.1093/hmg/ddt589 PMC3959811

[31] Cizmecioglu O, Ni J, Xie S, Zhao JJ, Roberts TM. Rac1-mediated membrane raft localization of PI3K/p110β is required for its activation by GPCRs or PTEN loss. eLife. 2016;5:e17635. 27700986 10.7554/eLife.17635 PMC5050018

[32] McPherson V, Reardon B, Bhayankara A, Scott SN, Boyd ME, Garcia-Grossman IR, et al. A phase 2 trial of buparlisib in patients with platinum-resistant metastatic urothelial carcinoma. Cancer. 2020;126(20):4532–44. 32767682 10.1002/cncr.33071 PMC8356147

[33] Takahashi N, Hao Z, Villaruz LC, Zhang J, Ruiz J, Petty WJ, et al. Berzosertib Plus Topotecan vs Topotecan Alone in patients with relapsed small cell lung cancer: a randomized clinical trial. JAMA Oncol. 2023;9(12):1669–77. 37824137 10.1001/jamaoncol.2023.4025 PMC10570917

[34] Middleton MR, Dean E, Evans TRJ, Shapiro GI, Pollard J, Hendriks BS, et al. Phase 1 study of the ATR inhibitor berzosertib (formerly M6620, VX-970) combined with gemcitabine ± cisplatin in patients with advanced solid tumours. Br J Cancer. 2021;125(4):510–9. 34040175 10.1038/s41416-021-01405-x PMC8368196

[35] Qiao Y, Zhu X, Li A, Yang S, Zhang J. Complete loss of STAG2 expression is an indicator of good prognosis in patients with bladder cancer. Tumor Biol. 2016;37(8):10279–86. 10.1007/s13277-016-4894-4 26838030

[36] Nie Z, Gao W, Zhang Y, Hou Y, Liu J, Li Z, et al. STAG2 loss-of-function mutation induces PD-L1 expression in U2OS cells. Ann Transl Med. 2019;7(7):127. 31157248 10.21037/atm.2019.02.23 PMC6511574

