# Supplementary material for: STAG2 expression imparts distinct therapeutic vulnerabilities in muscle-invasive bladder cancer cells
Source: Oncogenesis. 2025 Mar 1;14(1):4. doi: 10.1038/s41389-025-00548-3 (PMC11873148; doi:10.1038/s41389-025-00548-3)
Supplement: Supplementary file 1 — Supplemental Figures S1-S8, Tables S1 and S2, and Associated Legends [file 41389_2025_548_MOESM1_ESM.pdf]

Figure S1. Individual PI3K, MEK, and PLK inhibition across STAG2 intact and KO cell lines.

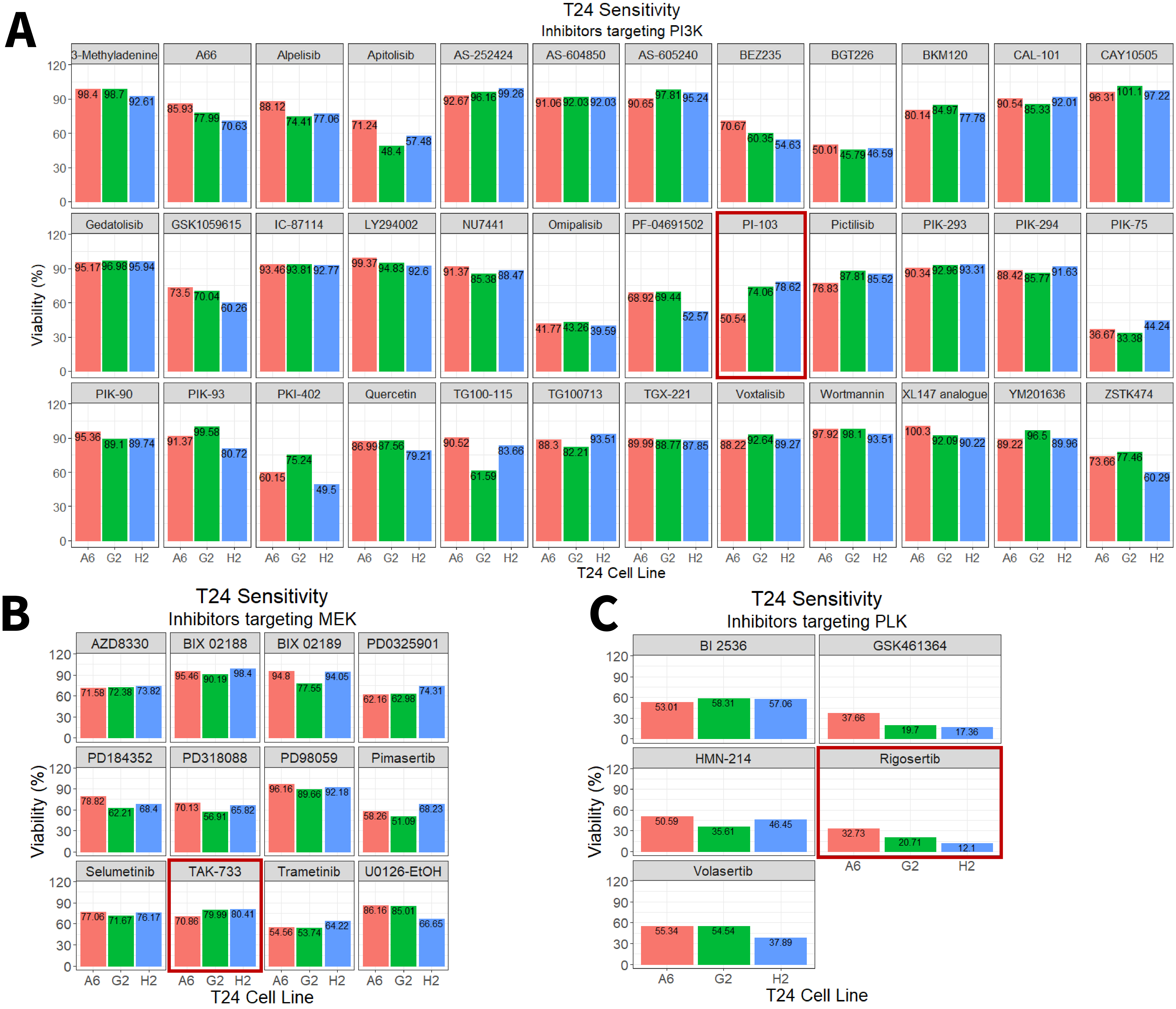

Figure S2. DepMap bladder cancer cell line sensitivity to candidate drugs grouped by STAG2 expression and p53 status.

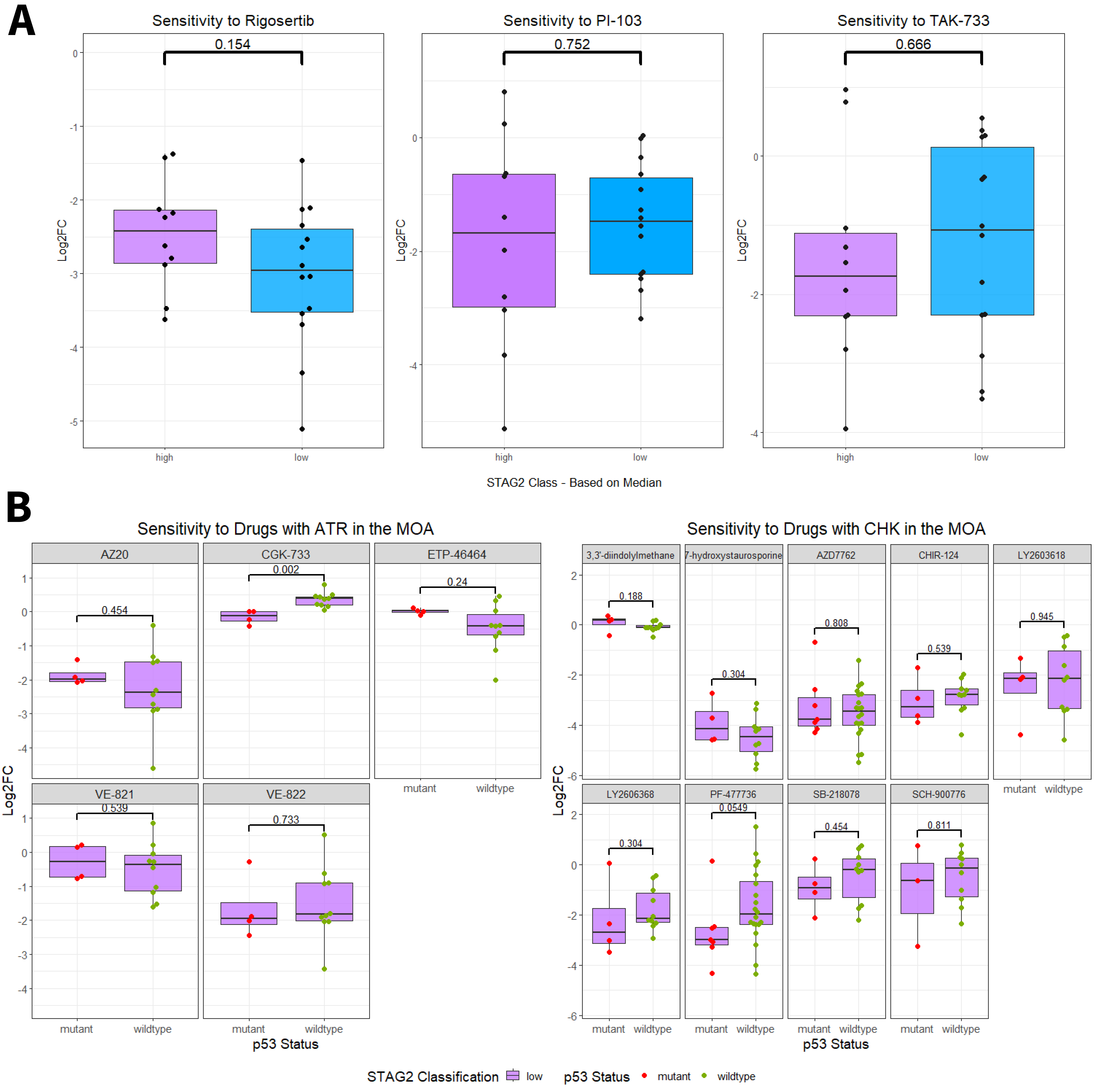

Figure S3. DepMap bladder cancer cell lines are similarly sensitive to PARP inhibition regardless of STAG2 expression.

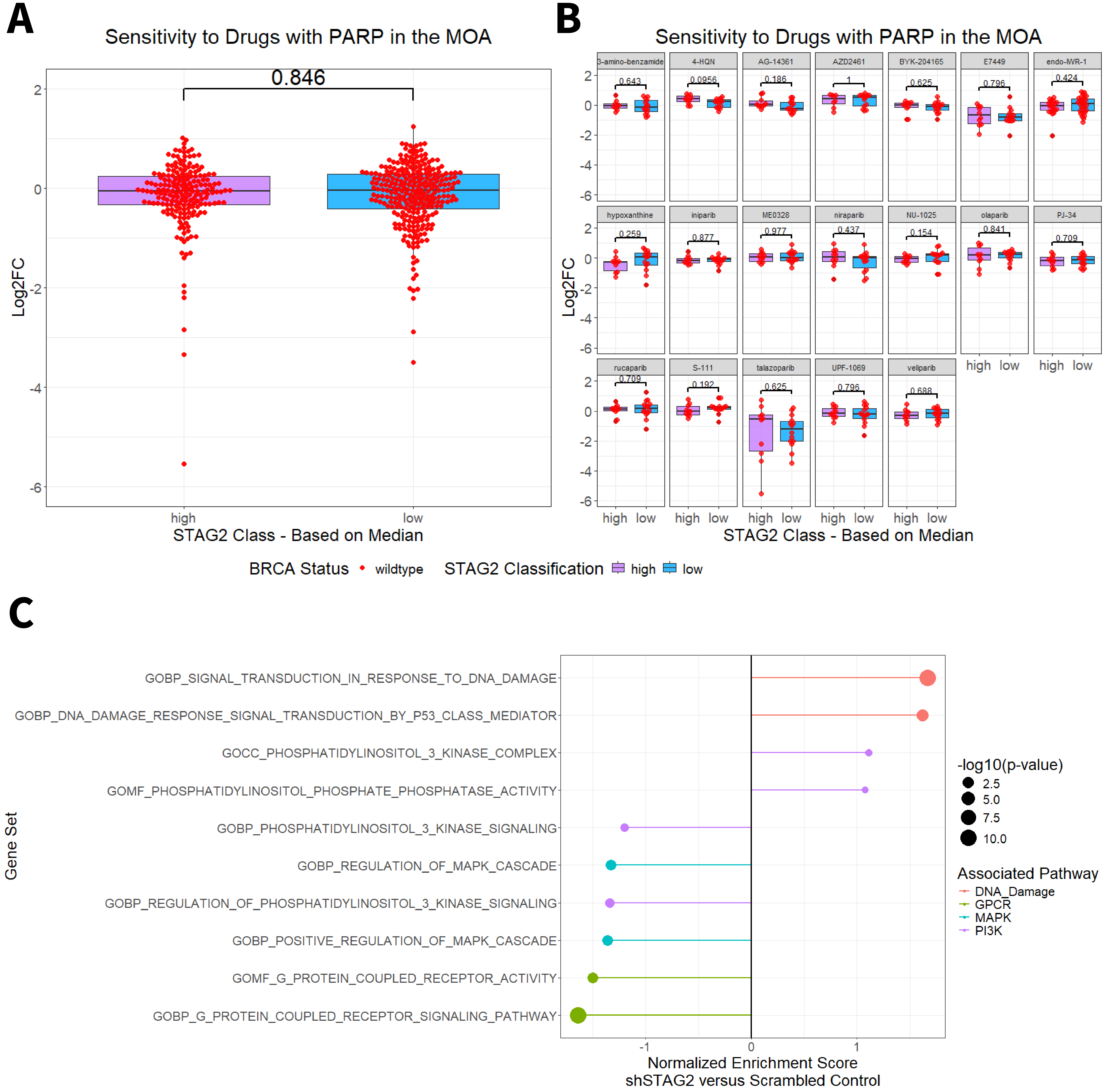

Figure S4. TCCSUP cells are equally sensitive to candidate drugs regardless of STAG2 status.

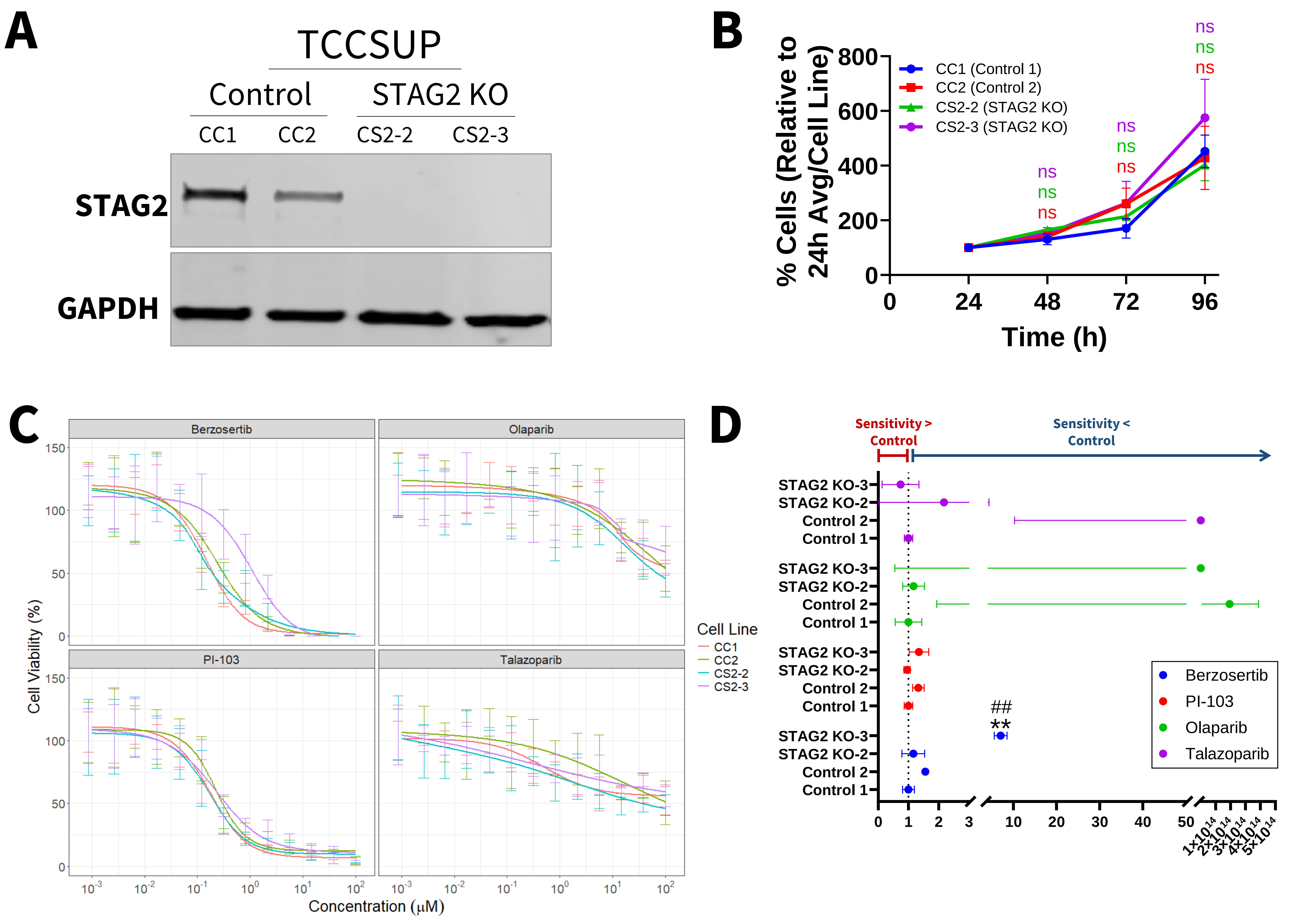

Figure S5. BO1 and HB-CLS-1 response to candidate drug treatment.

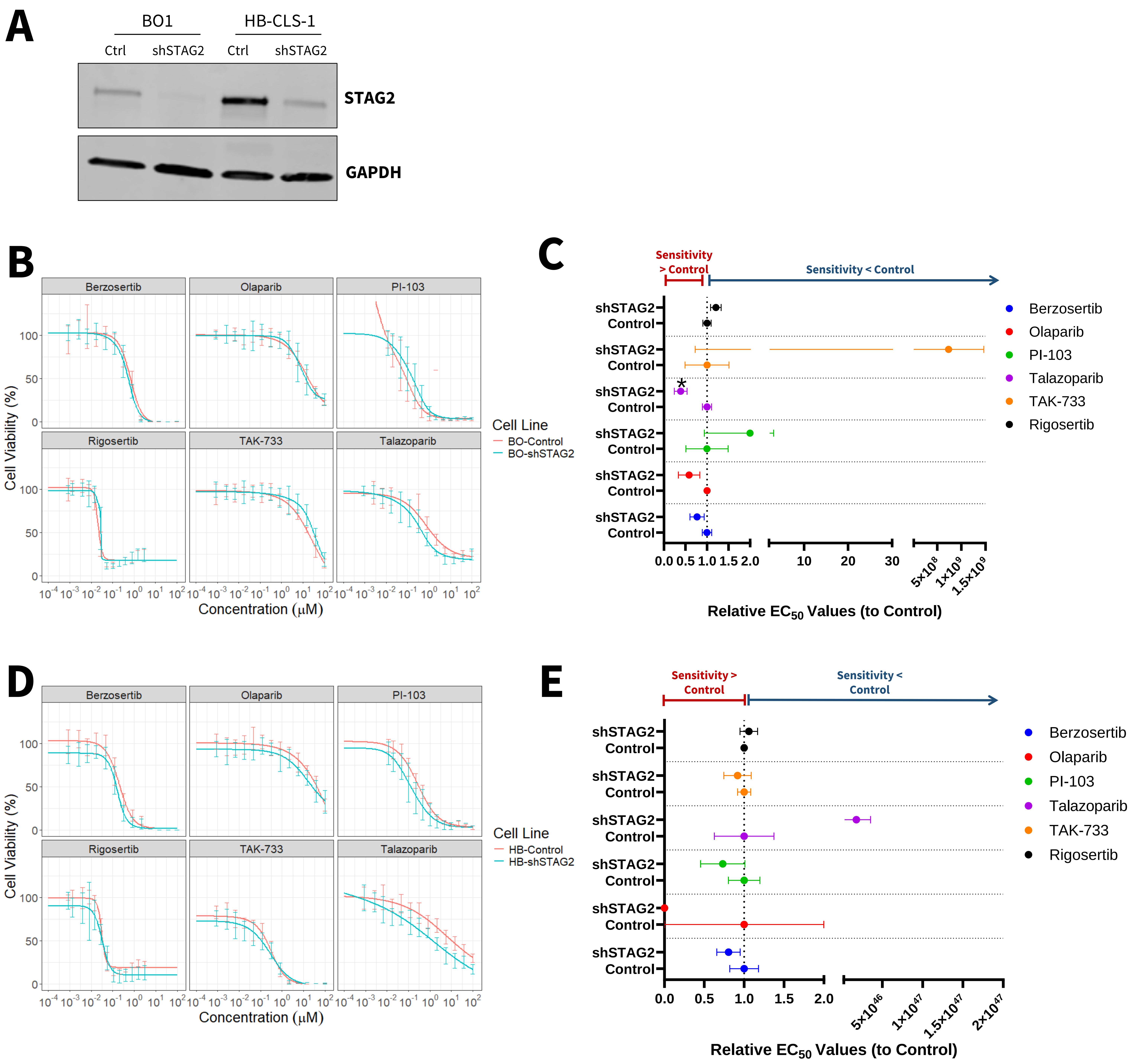

Figure S6. Dose response matrices for T24 cells treated with berzosertib, PI-103, olaparib, and talazoparib in combination with cisplatin.

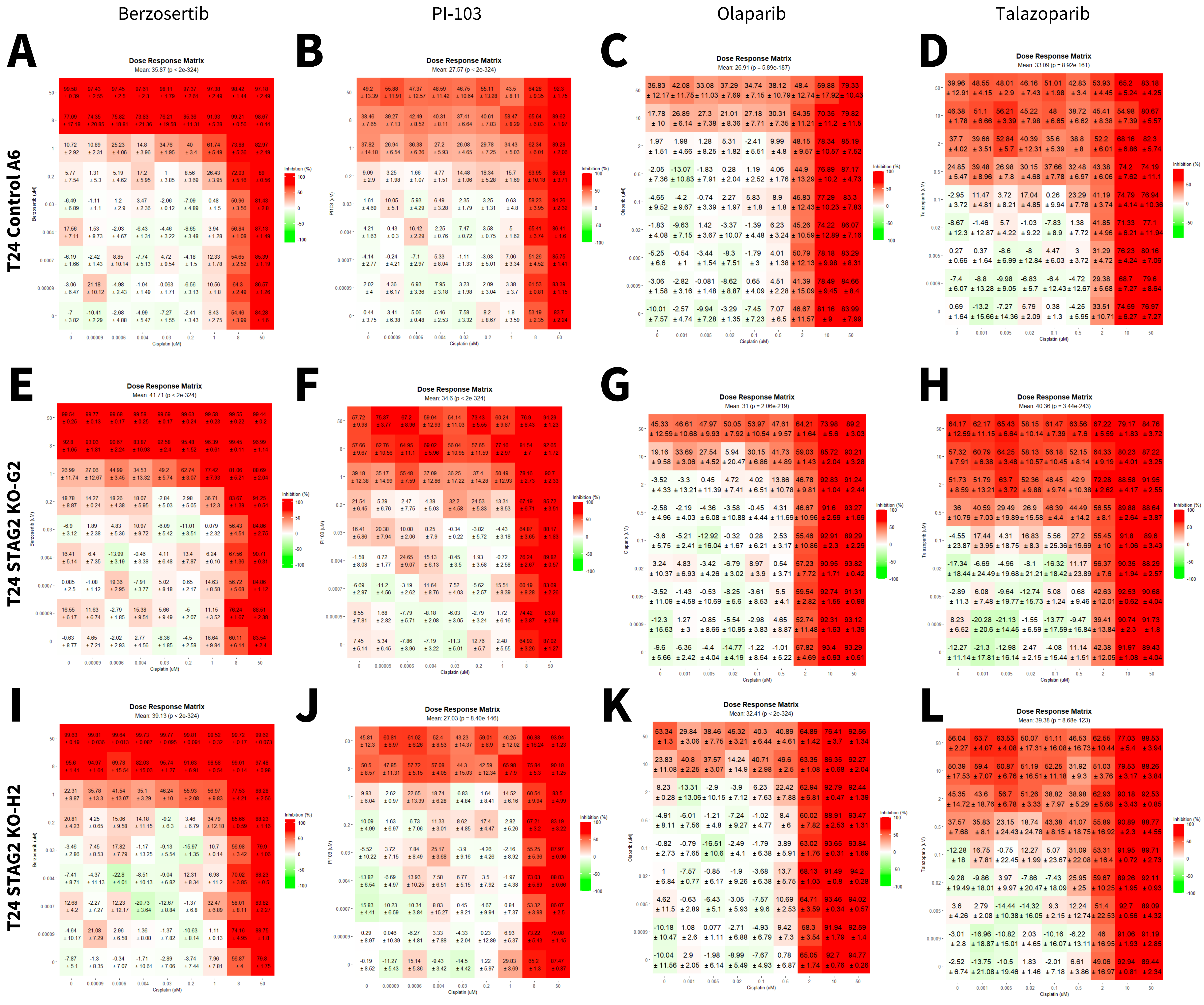

Figure S7. Dose response matrices for BO1 cells treated with berzosertib, PI-103, olaparib, and talazoparib in combination with cisplatin.

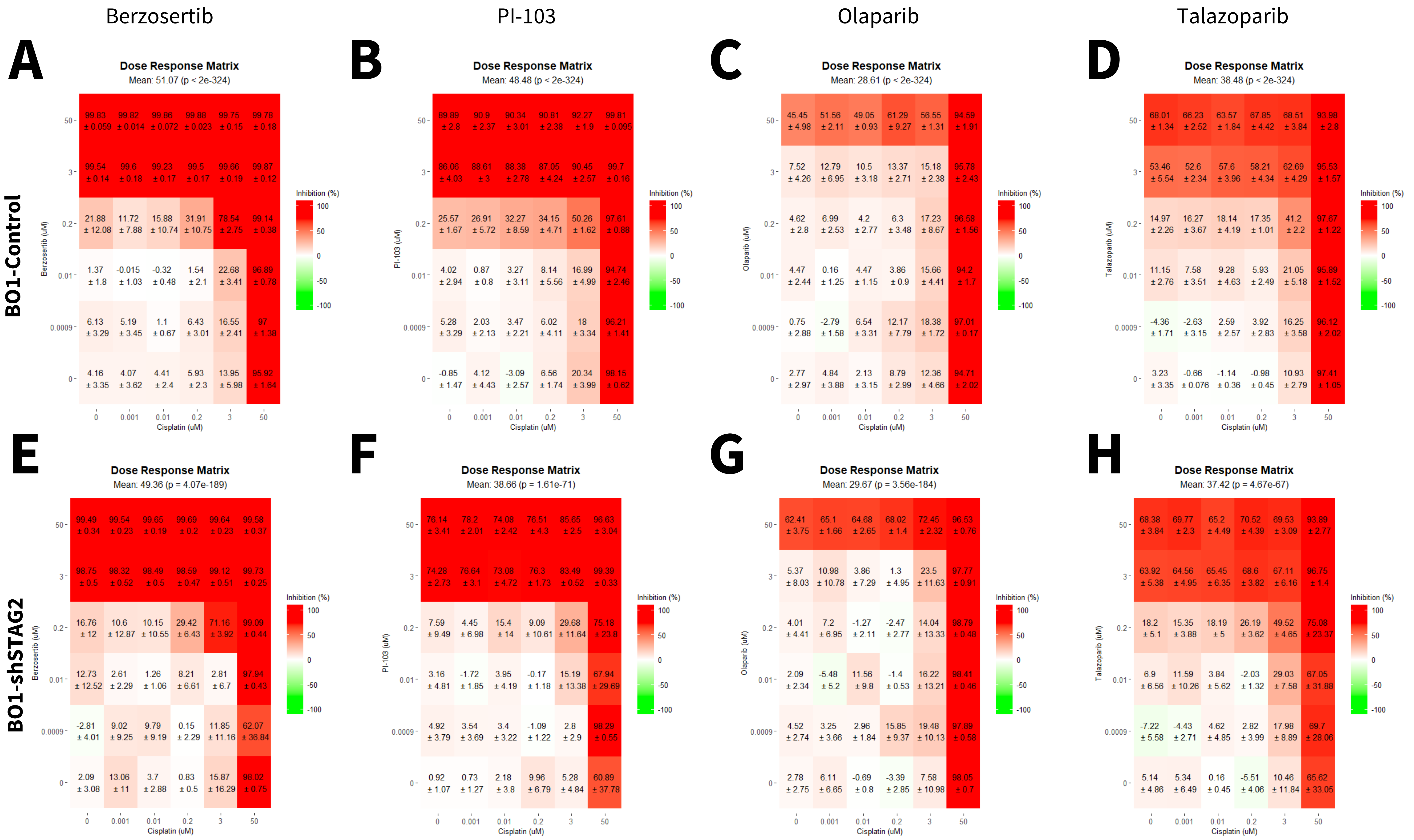

Figure S8. BO1 Synergy

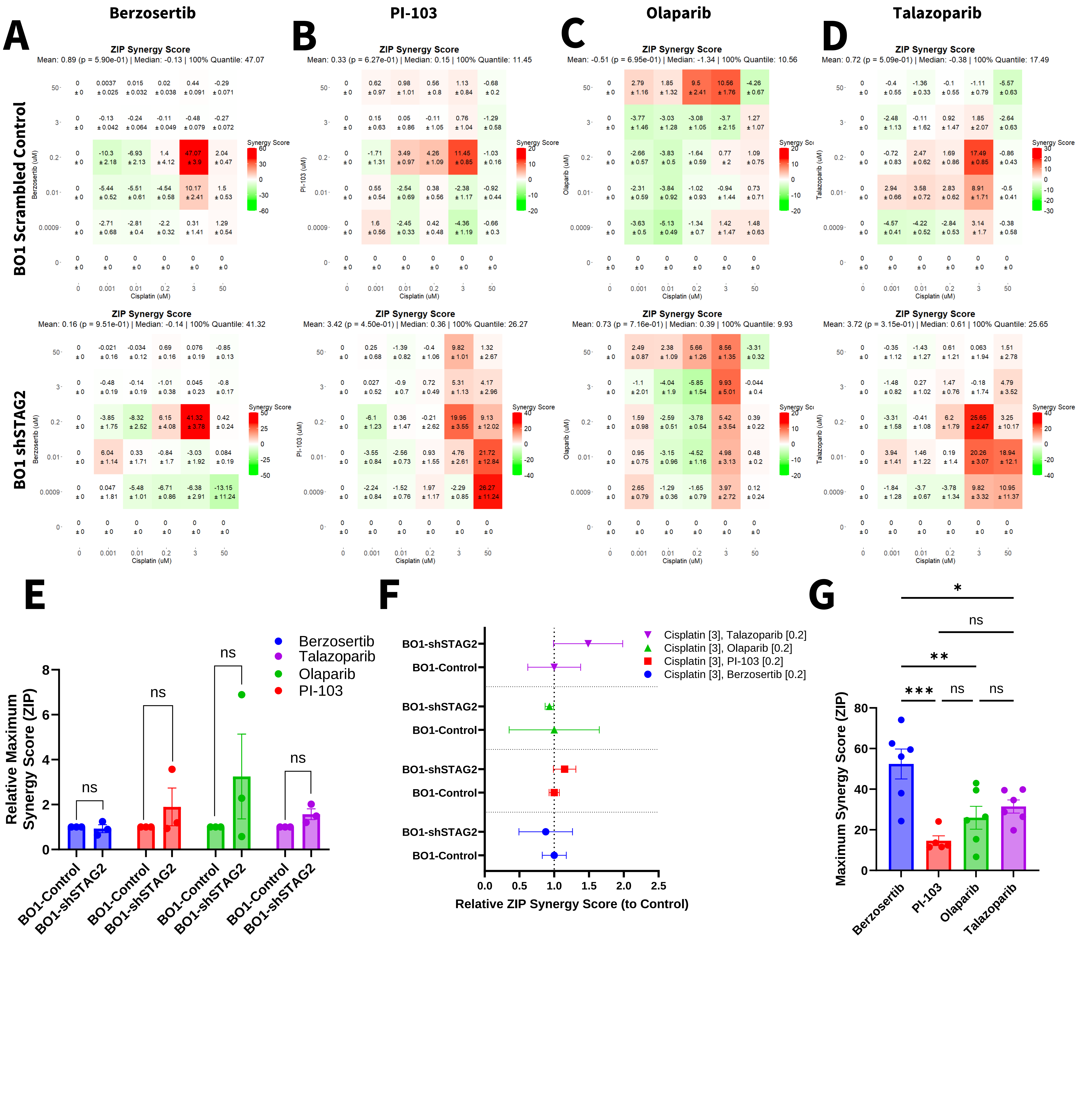

Table S1. All Drugs Included in Drug Screen

Drug Number	Drug	T24 A6 Viability (%)	T24 G2 Viability (%)	T24 H2 Viability (%)
1	ABT-263 (Navitoclax)	99.988	105.879	107.261
2	Roscovitine (Seliciclib,CYC202)	101.619	106.528	98.636
3	Nintedanib (BIBF 1120)	101.501	101.439	106.027
4	CYC116	100.250	102.070	103.633
5	Ridaforolimus (Deforolimus, MK-8669)	94.992	96.914	77.507
6	Irinotecan	77.909	84.991	69.407
7	Pazopanib HCl (GW786034 HCl)	99.691	108.668	104.325
8	Oxaliplatin	103.505	103.466	106.768
9	Trichostatin A (TSA)	86.744	88.900	73.370
10	Vemurafenib (PLX4032, RG7204)	100.915	108.161	102.925
11	Masitinib (AB1010)	100.029	111.283	104.902
12	Rigosertib (ON-01910)	40.431	30.310	22.591
13	SB202190 (FHPI)	102.270	108.880	110.469
14	Thiazovivin	106.884	106.990	108.450
15	GSK1904529A	105.081	108.675	103.687
16	PIK-93	94.886	104.762	86.643
17	OSU-03012 (AR-12)	101.774	111.757	104.391
18	Hesperadin	78.726	77.380	79.704
19	Palbociclib (PD-0332991) HCl	90.909	94.784	89.249
20	KRN 633	112.457	109.314	103.851
21	Pemetrexed	90.556	110.871	109.949
22	TWS119	100.034	103.274	101.306
23	BGJ398 (NVP-BGJ398)	105.581	102.837	104.933
24	Wortmannin	100.975	103.365	98.588
25	NVP-BHG712	97.427	110.924	104.162
26	CPI-613	98.588	107.050	104.276
27	SB590885	101.369	100.954	105.188
28	CEP-33779	99.619	98.956	101.586
29	Degrasyn (WP1130)	110.484	106.305	99.554
30	Tideglusib	98.439	106.460	100.902
31	TAK-733	75.844	86.270	86.353
32	WHI-P154	101.149	104.166	106.283
33	DCC-2036 (Rebastinib)	96.856	100.624	97.709
34	Birinapant	99.700	109.403	101.870
35	A-674563	97.594	102.729	97.941
36	CHIR-124	83.035	89.833	94.888
37	KX2-391	39.527	30.685	27.084
38	AG-1478 (Tyrphostin AG-1478)	100.977	102.838	105.210
39	CCT137690	95.447	100.393	93.966
40	ABT-737	99.405	107.089	95.130

41	SNS-314 Mesylate	88.957	90.699	91.292
42	Afatinib (BIBW2992)	96.739	98.602	102.072
43	WZ4002	99.919	100.248	98.552
44	Erlotinib HCl (OSI-744)	95.774	103.518	96.144
45	PIK-75	44.095	42.273	52.598
46	PD0325901	67.767	70.216	80.662
47	Etoposide	90.232	96.312	99.273
48	Vandetanib (ZD6474)	97.293	100.206	104.129
49	IC-87114	96.825	99.322	97.890
50	Pictilisib (GDC-0941)	81.391	93.654	91.125
51	Ki8751	92.802	102.024	97.697
52	MK-2206 2HCl	90.469	95.538	97.206
53	SP600125	98.400	99.680	101.108
54	PF-04217903	98.438	103.303	98.030
55	Ponatinib (AP24534)	73.267	88.352	69.014
56	Danusertib (PHA-739358)	99.330	101.004	101.942
57	BIX 02188	98.688	95.897	103.146
58	Triciribine	95.083	98.109	98.373
59	AT7867	102.439	108.969	101.507
60	Brivanib Alaninate (BMS-582664)	98.819	104.432	96.323
61	Gemcitabine	48.937	49.448	47.598
62	AST-1306	101.641	101.026	101.535
63	NVP-BVU972	99.082	101.513	102.235
64	OSI-420	93.366	96.972	96.147
65	AZD2014	69.512	68.761	71.862
66	Apatinib	97.096	93.122	103.529
67	Dabrafenib (GSK2118436)	70.287	70.934	72.277
68	BKM120 (NVP-BKM120, Buparlisib)	84.464	90.969	83.905
69	TPCA-1	92.099	100.468	94.878
70	LDN-193189	85.704	98.911	88.332
71	TG100713	92.040	88.368	98.581
72	CCT128930	98.004	96.236	98.885
73	Brefeldin A	24.625	26.575	29.671
74	AS-252424	96.096	101.538	103.949
75	NVP-BSK805 2HCl	93.988	92.471	99.900
76	GSK1838705A	99.203	95.848	95.721
77	SB415286	98.008	95.692	103.392
78	CHIR-98014	89.236	87.849	93.803
79	Linifanib (ABT-869)	98.552	104.652	100.177
80	MK-8245	87.221	90.924	92.038
81	Bosutinib (SKI-606)	91.434	88.312	84.003
82	PD98059	99.337	95.402	97.345
83	Gefitinib (ZD1839)	95.584	96.104	97.361
84	Tivozanib (AV-951)	88.754	94.216	99.547

85	PI-103	56.974	80.670	84.685
86	KU-0063794	81.104	84.182	86.581
87	Vorinostat (SAHA, MK0683)	89.284	75.207	93.373
88	BX-795	88.285	94.478	89.143
89	SB431542	95.660	102.308	101.004
90	Ruxolitinib (INCB018424)	97.644	84.624	98.208
91	PD153035 HCl	94.609	93.966	99.233
92	TSU-68 (SU6668, Orantinib)	100.744	96.721	85.042
93	MLN8054	83.836	75.816	91.612
94	LY2228820	86.813	91.691	89.300
95	TAE684 (NVP-TAE684)	84.390	64.545	76.325
96	BIX 02189	98.070	83.964	99.088
97	XL147 analogue	103.191	97.698	95.513
98	BMS-777607	95.895	101.149	89.752
99	AG-490 (Tyrphostin B42)	98.054	103.592	102.144
100	AICAR (Acadesine)	102.199	102.582	102.580
101	AZD8931 (Sapitinib)	102.478	101.526	98.305
102	Alectinib (CH5424802)	98.316	96.477	95.666
103	R788 (Fostamatinib) Disodium	83.971	96.049	87.216
104	TAK-285	93.200	98.422	93.394
105	TAME	93.037	88.255	98.410
106	Ipatasertib (GDC-0068)	94.207	97.226	85.288
107	CX-4945 (Silmitasertib)	94.622	88.756	84.375
108	Torin 1	56.404	53.538	58.406
109	AZD5438	64.109	80.086	76.775
110	IOX2	90.731	85.884	97.913
111	A66	89.832	84.382	77.229
112	EPZ5676	94.515	97.735	80.215
113	PF-00562271	83.883	76.771	76.778
114	WAY-600	78.177	85.013	89.478
115	TAK-901	93.071	90.033	92.624
116	Crenolanib (CP-868596)	97.640	93.348	84.450
117	AZ 628	93.882	85.938	93.259
118	Axitinib	99.977	106.334	101.946
119	Lenvatinib (E7080)	99.141	101.528	107.804
120	Cediranib (AZD2171)	96.406	94.248	98.401
121	Regorafenib (BAY 73-4506)	99.067	92.735	100.271
122	Imatinib Mesylate (STI571)	95.961	94.658	94.511
123	Doxorubicin (Adriamycin)	57.692	58.819	58.404
124	Rapamycin (Sirolimus)	64.884	87.376	74.268
125	AG-1024	93.847	93.110	97.700
126	VX-680 (Tozasertib, MK-0457)	77.436	84.113	81.530
127	BX-912	96.149	83.674	82.045
128	Crizotinib (PF-02341066)	89.168	94.699	87.900

129	Pelitinib (EKB-569)	56.974	75.680	67.418
130	SU11274	91.163	94.095	96.571
131	Pimasertib (AS-703026)	64.146	58.990	74.985
132	Vatalanib (PTK787) 2HCl	99.084	98.487	96.953
133	CCT129202	90.095	94.041	94.784
134	BI 2536	59.272	65.803	64.560
135	AZD7762	83.666	78.460	72.362
136	Cabozantinib (XL184, BMS-907351)	101.012	79.506	93.258
137	PD318088	75.169	64.487	72.734
138	SNS-032 (BMS-387032)	99.194	111.380	111.136
139	BMS-265246	71.548	94.594	76.715
140	GSK461364	45.017	29.363	27.508
141	3-Methyladenine (3-MA)	101.421	103.932	97.747
142	PIK-293	93.928	98.516	98.395
143	Capmatinib (INCB28060)	101.417	99.365	96.065
144	CAL-101 (Idelalisib, GS-1101)	94.121	91.312	97.179
145	INK 128 (MLN0128)	55.665	54.578	54.538
146	Indirubin	93.181	89.464	82.869
147	SAR131675	91.922	90.095	97.235
148	PP121	61.135	73.315	69.136
149	Piceatannol	96.389	83.636	98.788
150	NU7441 (KU-57788)	94.885	91.363	93.875
151	GSK J4 HCl	96.171	79.232	96.794
152	Trametinib (GSK1120212)	60.706	61.493	71.240
153	TG101209	88.147	89.049	94.791
154	AMG-900	81.274	77.423	79.088
155	Fedratinib (SAR302503, TG101348)	88.332	96.549	86.891
156	AMG-458	102.993	104.940	100.838
157	Saracatinib (AZD0530)	94.825	98.533	93.679
158	Cisplatin	98.492	100.692	100.556
159	Dovitinib (TKI-258, CHIR-258)	89.938	85.797	100.334
160	WZ8040	97.999	94.313	97.479
161	Lapatinib (GW-572016) Ditosylate	92.443	94.556	98.156
162	Fluorouracil (5-Fluoracil, 5-FU)	59.335	64.978	66.899
163	Sorafenib Tosylate	85.015	102.306	98.491
164	Amuvatinib (MP-470)	87.016	82.790	100.333
165	Y-27632 2HCl	98.948	92.326	83.161
166	AMG-208	93.491	96.158	89.351
167	PHA-665752	93.341	98.262	95.870
168	AS-605240	94.216	103.093	100.194
169	Brivanib (BMS-540215)	95.400	81.296	94.354
170	SB525334	97.739	100.160	102.999
171	U0126-EtOH	90.052	91.009	73.514
172	Voxtalisib (SAR245409, XL765)	91.966	98.217	94.626

173	Foretinib (GSK1363089)	90.608	85.303	79.759
174	R406 (free base)	78.978	68.859	73.550
175	Everolimus (RAD001)	84.824	82.458	69.999
176	KU-60019	95.385	97.048	86.340
177	Barasertib (AZD1152-HQPA)	95.783	102.437	96.158
178	AZD8330	76.512	79.085	80.207
179	R406	95.906	96.714	98.269
180	Dinaciclib (SCH727965)	56.897	50.859	56.794
181	AZ 960	91.358	99.222	95.607
182	Tofacitinib (CP-690550,Tasocitinib)	94.552	94.051	103.030
183	PIK-294	92.146	91.729	96.830
184	Alpelisib (BYL719)	91.869	81.008	83.232
185	Quercetin	90.820	93.414	85.239
186	Semaxanib (SU5416)	93.279	99.115	93.333
187	OSI-027	63.432	65.720	75.275
188	Entacapone	91.024	92.799	94.137
189	Omipalisib (GSK2126458, GSK458)	48.827	51.602	48.257
190	SGC 0946	97.782	73.079	86.672
191	Flavopiridol (Alvocidib) HCl	54.599	41.919	52.134
192	Resminostat	81.922	72.670	78.060
193	ZM 336372	92.763	90.248	89.826
194	PKI-402	65.899	81.791	57.504
195	BGT226 (NVP-BGT226)	56.478	53.991	54.791
196	Roxadustat (FG-4592)	99.000	109.746	101.024
197	CP-724714	97.505	100.867	99.535
198	Canertinib (CI-1033)	97.286	96.668	102.488
199	XAV-939	95.410	91.838	96.675
200	Panobinostat (LBH589)	21.671	28.279	26.084
201	PFI-1 (PF-6405761)	93.239	102.873	98.405
202	Sunitinib Malate	91.642	89.642	77.389
203	JNJ-7706621	76.595	75.835	81.546
204	Entinostat (MS-275)	74.317	73.984	78.243
205	TG100-115	94.096	68.904	89.386
206	ZSTK474	78.447	83.879	67.575
207	Staurosporine	11.035	11.393	12.655
208	NVP-ADW742	92.890	96.629	96.729
209	HMN-214	57.023	44.377	54.659
210	ZM 447439	91.395	84.519	83.367
211	AT7519	92.714	96.870	92.558
212	SGX-523	92.602	90.836	79.910
213	CP-673451	91.913	86.379	83.105
214	Mocetinostat (MGCD0103)	69.175	67.416	72.894
215	BS-181 HCl	98.667	87.837	95.000
216	Docetaxel	20.159	15.076	17.333

217	Neratinib (HKI-272)	92.463	95.391	95.990
218	A-966492	104.134	104.657	102.973
219	Dovitinib (TKI-258) Dilactic Acid	91.143	92.309	90.387
220	Mubritinib (TAK 165)	89.344	93.483	95.547
221	Sotrastaurin	93.345	93.559	97.788
222	Telatinib	95.211	94.510	95.954
223	Tyrphostin AG 879	100.432	89.451	90.790
224	Imatinib (STI571)	92.258	95.821	94.975
225	Baricitinib (LY3009104, INCB028050)	89.990	88.722	93.042
226	Fostamatinib (R788)	77.388	86.202	73.963
227	Tofacitinib (CP-690550) Citrate	95.904	89.803	97.226
228	WYE-125132 (WYE-132)	60.466	49.784	57.154
229	IWR-1-endo	95.912	81.090	76.849
230	Ibrutinib (PCI-32765)	94.127	89.397	90.652
231	Apitolisib (GDC-0980, RG7422)	76.195	56.448	64.954
232	PF-03814735	61.104	59.738	62.704
233	GSK1070916	84.956	88.222	78.808
234	Milciclib (PHA-848125)	63.956	62.734	63.392
235	Selumetinib (AZD6244)	81.596	78.414	82.396
236	TGX-221	93.607	94.564	93.301
237	PD184352 (CI-1040)	83.236	69.491	75.148
238	ENMD-2076	90.046	89.629	92.074
239	Motesanib Diphosphate (AMG-706)	93.088	96.005	92.478
240	YM201636	92.896	101.856	95.271
241	Tandutinib (MLN518)	93.370	94.466	94.820
242	PD173074	94.921	91.209	93.152
243	Enzastaurin (LY317615)	78.259	59.979	64.964
244	GSK1059615	78.296	76.884	67.551
245	SB216763	90.153	90.630	94.092
246	Aurora A Inhibitor I	90.618	96.862	96.901
247	OSI-906 (Linsitinib)	94.866	78.578	94.120
248	AEE788 (NVP-AEE788)	95.125	88.685	87.819
249	GDC-0879	95.686	95.305	93.554
250	MK-1775	67.643	66.858	68.962
251	GSK690693	101.401	92.790	99.235
252	AZD8055	55.240	54.596	59.377
253	Alisertib (MLN8237)	83.720	84.860	78.636
254	BIRB 796 (Doramapimod)	96.176	100.154	97.853
255	Paclitaxel	28.860	19.601	21.648
256	KW-2449	96.603	102.212	99.910
257	Raf265 derivative	102.106	101.715	103.304
258	MK-5108 (VX-689)	94.579	99.532	95.257
259	Torkinib (PP242)	76.740	84.666	75.794
260	WP1066	94.346	80.617	86.720

261	Volasertib (BI 6727)	61.430	62.251	46.665
262	Torin 2	57.977	57.430	51.938
263	Phenformin HCl	95.726	98.033	95.364
264	Golvatinib (E7050)	91.926	99.547	96.015
265	LY2603618	96.146	99.829	99.849
266	VX-702	96.541	90.086	101.996
267	ICG-001	101.084	93.125	100.214
268	3-deazaneplanocin A (DZNeP) HCl	94.784	93.052	91.856
269	AS-604850	94.598	97.641	97.198
270	A-769662	99.793	93.735	102.641
271	PH-797804	96.419	94.848	95.621
272	PHA-767491	94.252	96.286	95.656
273	Tivantinib (ARQ 197)	76.993	57.255	78.539
274	BEZ235 (NVP-BEZ235, Dactolisib)	75.666	67.731	62.292
275	WZ3146	93.761	98.495	99.782
276	Dasatinib	77.864	76.064	84.008
277	PIK-90	98.591	94.867	95.064
278	Nilotinib (AMN-107)	100.267	104.316	99.217
279	OSI-930	99.330	103.846	97.773
280	Temsirolimus (CCI-779, NSC 683864)	87.526	91.789	88.210
281	WYE-354	88.073	87.531	94.123
282	AC480 (BMS-599626)	102.152	96.597	100.086
283	MGCD-265	101.798	99.765	95.639
284	SB203580	93.796	103.278	99.879
285	PHA-680632	97.144	100.934	101.288
286	KU-55933 (ATM Kinase Inhibitor)	99.629	99.955	105.008
287	PHA-793887	101.227	98.364	104.620
288	LY294002	102.313	100.277	97.730
289	Quizartinib (AC220)	101.478	105.568	101.637
290	JNJ-38877605	96.277	101.007	100.041
291	PHT-427	101.200	100.483	105.909
292	AT9283	65.111	64.952	67.867
293	Tie2 kinase inhibitor	103.641	106.108	101.476
294	PLX-4720	95.239	110.660	102.538
295	Gandotinib (LY2784544)	96.576	100.322	99.663
296	BMS-794833	101.425	106.682	104.014
297	MK-2461	98.216	99.808	96.208
298	Momelotinib (CYT387)	90.957	100.059	96.512
299	AZD4547	98.538	103.000	101.260
300	Palomid 529 (P529)	94.703	86.557	96.416
301	TAE226 (NVP-TAE226)	94.699	91.508	97.811
302	Phenformin HCl	101.676	101.518	98.813
303	IMD 0354	84.362	95.920	89.248
304	Gedatolisib (PF-05212384, PKI-587)	98.419	102.313	100.848

305	WYE-687	93.411	90.451	92.051
306	BIX 01294	102.512	102.489	98.936
307	CAY10505	99.478	106.175	102.050
308	A-769662	103.219	98.854	110.384
309	Dacomitinib (PF299804, PF299)	99.950	100.933	97.485
310	PF-04691502	74.041	76.314	60.373
311	Varlitinib	107.267	103.098	106.049

Table S2. TCCSUP, BO1, and HB-CLS-1 EC50 values.

Drug	Cell Line	Condition	EC_{50} (μM) Mean	EC_{50} (μM) SEM
Berzosertib	TCCSUP	CRISPR Control 1 (CC1)	0.1512	0.0296
		CRISPR Control 2 (CC2)	0.2358	0.0139
		CRISPR STAG2 2 (CS2-3)	0.0175	0.0566
		CRISPR STAG2 3 (CS2-3)	1.0680	0.2241
PI-103	TCCSUP	CRISPR Control 1 (CC1)	0.1780	0.0251
		CRISPR Control 2 (CC2)	0.2357	0.0342
		CRISPR STAG2 2 (CS2-3)	0.1700	0.0180
		CRISPR STAG2 3 (CS2-3)	0.2399	0.0575
Olaparib	TCCSUP	CRISPR Control 1 (CC1)	20.8800	9.1920
		CRISPR Control 2 (CC2)	4.0330E+15	4.0330E+15
		CRISPR STAG2 2 (CS2-3)	24.3900	7.5330
		CRISPR STAG2 3 (CS2-3)	3.6390E+03	3628.0000
Talazoparib	TCCSUP	CRISPR Control 1 (CC1)	0.5232	0.0723
		CRISPR Control 2 (CC2)	256.4000	251.0000
		CRISPR STAG2 2 (CS2-3)	1.1360	1.1360
		CRISPR STAG2 3 (CS2-3)	0.3856	0.3194
Olaparib	BO1	Control	15.6247	9.0209
		shSTAG2	9.1337	5.2733
TAK-733	BO1	Control	138.2210	79.8019
		shSTAG2	1.0148E+11	5.8592E+10
Rigosertib	BO1	Control	0.0212	0.0122
		shSTAG2	0.0255	0.0147
Talazoparib	BO1	Control	0.7872	0.4545
		shSTAG2	0.3060	0.1767
PI-103	BO1	Control	0.0861	0.0497
		shSTAG2	0.1721	0.0993
Berzosertib	BO1	Control	0.6781	0.3915
		shSTAG2	0.5210	0.3008
Olaparib	HB-CLS-1	Control	1.8726E+06	1.5287E+06
		shSTAG2	22.1924	8.6560
TAK-733	HB-CLS-1	Control	0.2991	0.0199
		shSTAG2	0.2742	0.0421
Rigosertib	HB-CLS-1	Control	0.0306	0.0004
		shSTAG2	0.0324	0.0027
Talazoparib	HB-CLS-1	Control	10.2363	3.1304
		shSTAG2	1.8087E+47	1.4768E+47
PI-103	HB-CLS-1	Control	0.2777	0.0446
		shSTAG2	0.2029	0.0630
Berzosertib	HB-CLS-1	Control	0.2105	0.0311
		shSTAG2	0.1692	0.0253

Supplemental Figure Legends

Figure S1. Individual PI3K, MEK, and PLK inhibition across STAG2 intact and KO cell lines. Viability of T24 A6 (Control), G2 (STAG2 KO), and H2 (STAG2 KO) cell lines after 72 hours of treatment with all individual drugs targeting **A.** PI3K, **B.** MEK, and **C.** PLK in the custom drug screen. Numbers in each bar represent viability of the associated cell line. Red box indicates one drug from each category that was chosen for further testing (PI3K: PI-103; MEK: TAK-733; PLK: Rigosertib).

Figure S2. DepMap bladder cancer cell line sensitivity to candidate drugs grouped by STAG2 expression and p53 status. A. Log2FC of STAG2 high and STAG2 low DepMap bladder cancer cell lines after treatment with TAK-733 (left), Rigosertib (middle) and PI-103 (right) relative to DMSO control treatment. P-values calculated with two sample Wilcoxon test. Log2FC: Log2 fold change. Boxplots indicated first quartile, median, and third quartile. **B.** Log2FC of STAG2 low DepMap bladder cancer cell lines after treatment with individual inhibitors targeting CHK (right) or ATR (left), relative to DMSO control. Red indicates cell lines that are p53 mutant, green indicates cell lines that are p53 wildtype. P-values calculated by two sample Wilcoxon test. MOA: Mechanism of action; Log2FC: Log2 fold change; CHK: checkpoint kinase; ATR: ataxia telangiectasia and Rad3-related.

Figure S3. DepMap bladder cancer cell lines are similarly sensitive to PARP inhibition regardless of STAG2 expression. A. Log2FC of STAG2 high and STAG2 low DepMap bladder cancer cell lines after treatment with drugs that include PARP in their mechanism of action. An increasingly negative Log2FC indicates increasing sensitivity relative to DMSO control. **B.** Log2FC of STAG2 high and STAG2 low DepMap bladder cancer cell lines after treatment with individual PARP inhibitors. All cell lines were BRCA wild type, as indicated by the red color. P-values calculated with two sample Wilcoxon test. MOA: Mechanism of action; Log2FC: Log2 fold change; PARP: Poly (ADP-ribose) polymerase. **C.** Normalized enrichment scores (NES) of gene ontology (GO) pathways analyzed through Gene Set Enrichment Analysis (GSEA) of RNA-sequencing data. T24 cells with shRNA mediated STAG2 knockdown were compared to

control T24 cells. Positive NES indicates pathways enrichment in STAG2 KD, negative NES indicates pathway enrichment in STAG2 WT.

Figure S4. TCCSUP cells are equally sensitive to candidate drugs regardless of STAG2 status. A. Western blot of isogenic clonal TCCSUP cell lines indicating complete knock out of STAG2 protein expression in STAG2 KO CS2-2 and STAG2 KO CS2-3 cell lines. Control CC1 and control CC2 cell lines maintain STAG2 expression. GAPDH used as a loading control. **B.** Cell number of TCCSUP control CC1 and CC2, and STAG2 KO CS2-2 and CS2-3 cell lines over a period of 96 hours measured via SRB assay every 24 hours. Data is representative of three independent experiments and a minimum of five technical replicates. Presented as % cells relative to the average at 24 hours for each cell line. Error bars represent SEM. Statistics assessed via one-way ANOVA with multiple comparisons using Control CC1 as a reference. **C.** Dose response curves for TCCSUP control CC1 (red), control CC2 (green), STAG2 KO CS2-2 (blue), and STAG2 KO CS2-3 (purple) cell viability after treatment with the indicated candidate drugs and PARP inhibitors olaparib and talazoparib at doses ranging from 10^{-4} μM to 10^{2} μM for 72 hours. Curves modeled using package drda in R; representative of at least three independent experiments. Error bars represent standard deviation. Cell viability normalized and calculated as a percentage compared to untreated wells within each individual experiment. **D.** Relative EC_{50} values for TCC control 1 (CC1), control 2 (CC2), STAG2 KO-2 (CS2-2), and STAG2 KO-3 (CS2-3) cell lines derived from three independent experiments as displayed in S4C. A relative EC_{50} value greater than one indicates that the cell line has a higher EC_{50} than control and therefore is less sensitive to the drug. A relative EC_{50} value lower than one indicates that the cell line has a lower EC_{50} than control, and therefore is more sensitive to the drug. Statistical significance analyzed via t-test; *$p<0.05$ compared to control 1 cell line; #$p<0.05$ compared to control 2 cell line.

Figure S5. BO1 and HB-CLS-1 response to candidate drug treatment. A. Western blot of STAG2 and GAPDH expression in BO1 and HB-CLS-1 cell lines engineered to express a scrambled control (Ctrl) shRNA or shRNA targeting STAG2 (shSTAG2). GAPDH used as a loading control. **B.** Dose response curves for BO1 control (red) and BO1 shSTAG2 (blue) cell lines after treatment with the indicated candidate

drugs at doses ranging from 10^{-3} μM to 10^{2} μM for 72 hours. Cell viability normalized and calculated as a percentage compared to untreated wells within each individual experiment. **C.** Relative EC_{50} values for BO1 Control and shSTAG2 cell lines as displayed in S5B. A relative EC_{50} value greater than one indicates that the cell line has a higher EC_{50} than control and therefore is less sensitive to the drug. A relative EC_{50} value lower than one indicates that the cell line has a lower EC_{50} than control, and therefore is more sensitive to the drug. Statistical significance analyzed via t-test; *$p<0.05$. **D.** Dose response curves for HB-CLS-1 control (red) and BO1 shSTAG2 (blue) cell lines after treatment with the indicated candidate drugs at doses ranging from 10^{-3} μM to 10^{2} μM for 72 hours. **E.** Relative EC_{50} values for HB-CLS-1 Control and shSTAG2 cell lines as displayed in **S5D**. A relative EC_{50} value greater than one indicates that the cell line has a higher EC_{50} than control and therefore is less sensitive to the drug. A relative EC_{50} value lower than one indicates that the cell line has a lower EC_{50} than control, and therefore is more sensitive to the drug. Statistical significance analyzed via t-test; ****$p<0.0001$. Dose response curves modeled using package drda in R; representative of at least three independent experiments. Cell viability normalized and calculated as a percentage compared to untreated wells within each individual experiment. Dose response curve error bars represent standard deviation of cell viability at each concentration; relative EC_{50} values represented as mean ± SEM.

Figure S6. Dose response matrices for T24 cells treated with berzosertib, PI-103, olaparib, or talazoparib in combination with cisplatin. A-D. Dose response viability matrices for berzosertib (A), PI-103 (B), olaparib (C), or talazoparib (D) in combination with cisplatin in T24 A6 (STAG2 WT) cell line. **E-H.** Dose response viability matrices for berzosertib (E), PI-103 (F), olaparib (G), or talazoparib (H) in combination with cisplatin in T24 G2 (STAG2 KO) cell line. **I-L.** Dose response viability matrices for berzosertib (I), PI-103 (J), olaparib (K), or talazoparib (L) in combination with cisplatin in T24 G2 (STAG2 KO) cell line. Values plotted as % inhibition, red indicates decreased viability after treatment, green indicates increased viability after treatment. All viability values displayed as mean ± SEM.

Figure S7. Dose response matrices for BO1 cells treated with berzosertib, PI-103, olaparib, and talazoparib in combination with cisplatin. A-D. Dose response viability matrices for berzosertib (A), PI-103 (B), olaparib (C), or talazoparib (D) in combination with cisplatin in BO1 Control (STAG2 WT) cell line. **E-H.** Dose response viability matrix for berzosertib (E), PI-103 (F), olaparib (G), or talazoparib (H) in combination with cisplatin in BO1 shSTAG2 cell line. Values plotted as % inhibition, red indicates decreased viability after treatment, green indicates increased viability after treatment. All viability values displayed as mean ± SEM.

Figure S8. Berzosertib, PI-103, olaparib, and talazoparib exhibit synergy in combination with cisplatin in BO1 cells. 2 drug combination matrices of ZIP synergy scores for the combination of **A.** berzosertib, **B.** PI-103, **C.** Olaparib, **D.** or talazoparib with cisplatin calculated using the ZIP synergy model for BO1 Control cells (top row) and BO1 shSTAG2 cells (bottom row). 100% quantile represents the maximum synergy score achieved in each cell line for the combination. **E**. Ratio of the maximum synergy score for BO1 shSTAG2 cells relative to BO1 Control cells. **F**. Ratio of the synergy score for each cell line relative to BO1 control cells at the specified concentrations (µM) of cisplatin and indicated candidate drug. A synergy ratio greater than one indicates that the drug combination was more synergistic in the indicated cell line compared to control. A synergy ratio less than one indicates that the drug combination was less synergistic in the indicated cell line compared to control. Statistical comparisons analyzed via t-test; *$p<0.05$. **G**. Maximum ZIP synergy scores of indicated drugs in combination with cisplatin in all T24 cell lines combined. Statistical differences analyzed via one-way ANOVA with multiple comparisons. *$p<0.05$; **$p<0.01$.

Supplemental Table Legends

Table S1. All drugs included in screen. All drugs tested in drug screen in T24 cells as described in Figure 1. T24 cell lines (A6, STAG2 WT/Control; G2, STAG2 KO; H2, STAG2 KO) were treated with each of the indicated drugs for 72 hours and analyzed for cell viability.

Table S2. TCCSUP, BO1 and HB-CLS-1 EC_{50} values. Average and SEM of EC_{50} values (μM) calculated from dose response curves for each drug tested in TCCSUP, BO1, and HB-CLS-1 cell lines. Each EC_{50} is representative of three independent experiments.
